# Increased Expression and Altered Cellular Localization of Fibroblast Growth Factor Receptor-Like 1 (FGFRL1) Are Associated with Prostate Cancer Progression

**DOI:** 10.3390/cancers14020278

**Published:** 2022-01-07

**Authors:** Lan Yu, Mervi Toriseva, Syeda Afshan, Mario Cangiano, Vidal Fey, Andrew Erickson, Heikki Seikkula, Kalle Alanen, Pekka Taimen, Otto Ettala, Martti Nurmi, Peter J. Boström, Markku Kallajoki, Johanna Tuomela, Tuomas Mirtti, Inès J. Beumer, Matthias Nees, Pirkko Härkönen

**Affiliations:** 1 Institute of Biomedicine and FICAN West Cancer Centre, University of Turku and Turku University Hospital, 20520 Turku, Finland; yulan@zzu.edu.cn (L.Y.); mertor@utu.fi (M.T.); syeda.afshan@utu.fi (S.A.); vidal.fey@tuni.fi (V.F.); pepeta@utu.fi (P.T.); matthias.nees@utu.fi (M.N.); 2 GenomeScan, 2333 BZ Leiden, The Netherlands; mario.cangiano@biogemcampus.com (M.C.); i.beumer@genomescan.nl (I.J.B.); 3 Nuffield Department of Surgical Sciences, University of Oxford, Oxford 0X3 9DU, UK; andrew.erickson@helsinki.fi; 4 Department of Urology, University of Turku and Turku University Hospital, 20520 Turku, Finland; heikki.seikkula@ksshp.fi (H.S.); otto.ettala@tyks.fi (O.E.); Martti.nurmi@fimnet.fi (M.N.); peter.j.bostrom@gmail.com (P.J.B.); 5 Department of Pathology, Turku University Hospital, 20520 Turku, Finland; kalle.a.alanen@gmail.com (K.A.); markku.kallajoki@tyks.fi (M.K.); 6 HUS Diagnostic Center and Research Program in Systems Oncology (ONCOSYS), Helsinki University Hospital and University of Helsinki, 00014 Helsinki, Finland; tuomas.mirtti@helsinki.fi; 7 Department of Biochemistry and Molecular Biology, Medical University in Lublin, 20-093 Lublin, Poland

**Keywords:** prostate cancer, FGFRL1 (FGFR5), FGFR signaling, tumor–stromal interactions, biochemical recurrence, prostate cancer progression

## Abstract

**Simple Summary:**

Prostate cancer (PCa) is one of the most frequently diagnosed malignancies in men. PCa is primarily regulated by androgens, but other mechanisms, such as fibroblast growth factor receptor (FGFR) signaling, are also involved. In some patients, PCa relapses after surgical removal of prostate, and androgen deprivation therapy (ADT) is used as the first-line treatment. Unfortunately, the patients often lose response to ADT and progress by other mechanisms to castration-resistant, currently non-curable PCa. In our study, we aimed to identify better diagnostic markers and therapeutic targets against PCa. We analyzed patient PCa tissue samples from radical prostatectomies and biopsies, and used physiologically relevant 3D organoids and mouse xenografts to study FGFR signaling in PCa. We found that FGFRL1, a protein belonging to the FGFR family, plays a role in PCa. Our results suggest that FGFRL1 has significant effects on PCa progression and has potential as a prognostic biomarker.

**Abstract:**

Fibroblast growth factor receptors (FGFRs) 1–4 are involved in prostate cancer (PCa) regulation, but the role of FGFR-like 1 (FGFRL1) in PCa is unclear. FGFRL1 expression was studied by qRT-PCR and immunohistochemistry of patient tissue microarrays (TMAs) and correlated with clinical patient data. The effects of FGFRL1 knockdown (KD) in PC3M were studied in in vitro culture models and in mouse xenograft tumors. Our results showed that FGFRL1 was significantly upregulated in PCa. The level of membranous FGFRL1 was negatively associated with high Gleason scores (GSs) and Ki67, while increased cytoplasmic and nuclear FGFRL1 showed a positive correlation. Cox regression analysis indicated that nuclear FGFRL1 was an independent prognostic marker for biochemical recurrence after radical prostatectomy. Functional studies indicated that FGFRL1-KD in PC3M cells increases FGFR signaling, whereas FGFRL1 overexpression attenuates it, supporting decoy receptor actions of membrane-localized FGFRL1. In accordance with clinical data, FGFRL1-KD markedly suppressed PC3M xenograft growth. Transcriptomics of FGFRL1-KD cells and xenografts revealed major changes in genes regulating differentiation, ECM turnover, and tumor–stromal interactions associated with decreased growth in FGFRL1-KD xenografts. Our results suggest that FGFRL1 upregulation and altered cellular compartmentalization contribute to PCa progression. The nuclear FGFRL1 could serve as a prognostic marker for PCa patients.

## 1. Introduction

Prostate cancer (PCa) is one of the most frequently diagnosed malignancies in men, with an estimated 1.3 million new cases worldwide in 2018 [1]. Radical prostatectomy (RP) is an effective treatment for patients with localized PCa, typically supporting long-term progression-free survival; however, around 30% of PCa patients relapse within 10 years. PCa is regulated by androgens and androgen receptor (AR), which is the basis for the treatment of relapsed patients with androgen deprivation therapy (ADT). Over 20% of these patients develop aggressive castration-resistant prostate cancer (CRPC) and fail to benefit from ADT. The mechanisms leading to CRPC are not fully understood, and reliable prognostic markers and effective treatments are currently unknown [2].

Other mechanisms, including fibroblast growth factors (FGFs) and fibroblast growth factor receptors (FGFRs), also participate in PCa development and progression [3,4,5,6]. FGFs, FGFRs, and downstream signaling pathways regulate various cellular functions, such as proliferation, differentiation, morphogenesis, apoptosis, migration, and angiogenesis [3,4,7,8]. The primary feature of FGF/FGFR actions is the control of reciprocal signaling between epithelial and stromal compartments in developmental processes, organ homeostasis, angiogenesis, and the repair of wounded and damaged tissues [4,5]. Increased oncogenic signaling of the FGF/FGFR pathways by activating gene mutations, amplifications, and fusions; overexpression of FGFR protein; or altered FGF ligand production and autocrine/paracrine signaling may be involved in the initiation and progression of different types of cancer, including PCa [3,4,7,9,10,11,12,13].

FGFRL1 is the fifth and most recently identified FGFR [14]. Its extracellular domain is very similar to FGFR1-4 and binds FGFs, such as FGF-2 and FGF-8, with high affinity. In contrast to FGFR1-4, the intracellular tyrosine kinase domain of FGFRL1 is replaced by a short histidine-rich C-terminal tail, which is unable to convey canonical signal transduction through receptor auto-phosphorylation [14]. Fgfrl1-deficient mice die at birth and show multiple congenital malformations, such as hypoplastic diaphragms or dysfunctional metanephric kidneys [15,16]. Mice lacking the FGFRL1 intracellular domain are viable and develop normally. This domain may regulate the turnover of the FGFRL1 extracellular domain [15]. It also binds to SHP-1 phosphatase in pancreatic islet beta cells, which may affect cellular signaling [17] and negatively acting Spread 1, a member of the Sprouty/Spread family [18]. Due to a truncated intracellular domain, FGFRL1 was originally assumed to primarily act as a decoy receptor for FGF ligands [19,20]. However, later studies have also suggested other mechanisms, such as the function of FGF8-bound FGFRL1 as a coreceptor via FGFR1 in the regulation of nephron development [21]. 

FGFRL1 is widely expressed in human tissues [15,22]. In mesenchymal cells, FGFRL1 inhibits cell proliferation [22], is associated with cell differentiation [22,23], and induces cell adhesion [24,25]. Increased expression of FGFRL1 in ovarian cancers [26] correlates with poor prognoses for ovarian cancer patients [27]. In esophageal squamous cell carcinoma (ESCC) cells, FGFRL1 deficiency decreases tumor growth in xenografts [28], and its expression is increased in clinical ESCC tumors [29]. In addition to altered expression, genetic deletions and mutations have been demonstrated in bladder and colon cancer, respectively [30,31]. 

Little is known about FGFRL1 in PCa, although FGF/FGFR pathways have an established role in prostate development and functioning [4,5,7,8,10]. In this study, we analyzed FGFRL1 expression in PCa using publicly available mRNA gene expression data sets and examined mRNA levels and protein expression in benign prostate and PCa tissue samples from radical prostatectomies and biopsies of PCa patients in our study cohorts. Immunohistochemically detected FGFRL1 protein expression and cellular localization in TMAs were scored and correlated with clinicopathological parameters and patient follow-up data (time to BCR). The functions of FGFRL1 were studied using PC3M KD cell models with control cell lines in 2D and 3D cultures and xenografts. The gene expression profiles of FGFRL1-KD cell lines and xenografts compared to controls were studied with RNA-seq analyses. 

## 2. Materials and Methods

### 2.1. Gene Expression Database Mining

Expression and correlation data for FGFRL1 were extracted from a clinical transcriptome study (MSKCC 2010) [32], available through the cBioPortal database (http://www.cbioportal.org/public-portal, 1 April 2017) [33]. The database contains 218 clinical PCa samples and metastases, cell lines, and normal prostate tissues [32]. The mRNA gene expression data were extracted, normalized, and analyzed using an in-house HTML interface (REX) with R/bioconductor-based algorithms. Associations of median-centered gene expression patterns with clinical annotations (grades, GSs, and TNM staging) and selected clinicopathological features (e.g., extracapsular invasion) were analyzed and plotted using R/bioconductor-based algorithms. Additionally, the Medisapiens IST data sets for multiple PCa studies were analyzed and plotted in a similar manner [34].

### 2.2. Patient Data and Tissue Microarray (TMA) Construction

Primary PCa samples from patients undergoing RP and pelvic lymph node dissection, and biopsy samples from locally invasive and metastatic advanced prostate cancer (AdvPCa) tumors, were collected between 1993 and 2011 at the Turku University Hospital TYKS [35]. Transurethral resections of prostate specimens were searched in the Helsinki University Hospital pathology archive for 2007–2011. Additionally, nonmalignant prostate tissue samples from patients undergoing surgery for benign prostatic hyperplasia (BPH) were included in the study. Fresh samples of primary PCa and nonmalignant adjacent prostate (AdjPr) tissues were frozen for RNA extraction. Parallel samples were fixed in formalin, embedded in paraffin, and sectioned for histopathological analysis. Biopsy samples from AdvPCa and BPH were processed similarly for histopathology and screened to determine their CRPC statuses. Clinical pathologists (T.M., K.A., M.K., or P.T.) examined all the H&E-stained sections and confirmed the Gleason score (GS) [36,37] of the PCa sample areas and the CRPC statuses of the transurethral resection specimens used for constructing the following TMAs. 

TMA I comprised BPH (*n* = 5) nonmalignant tissues adjacent to AdjPr (*n* = 189) and high-grade prostatic intraepithelial neoplasia (HGPIN) (*n* = 35) tissues, and primary PCa tissues from patient samples of sufficient size and in proportions of PCa > 50% of the sample area (*n* = 144) [35]. 

TMA II comprised 62 biopsies from 36 patients with AdvPCa (19 with seminal vesicle invasion, 35 with pelvic lymph node metastasis, 4 with bone metastasis, and 4 with distant tissue metastasis).

TMA III comprised transurethral resection samples from CRPC patients (*n* = 21). 

The TMA blocks were constructed as described by Yu et al. [35]. The sectioning of the TMA blocks, immunostaining of FGFRL1 and Ki67, and evaluation and scoring of the membranous, cytoplasmic, and nuclear immunostaining for FGFRL1 are described in the Supplementary Materials and Methods file.

### 2.3. Collection and Statistical Analysis of Clinical Data

The inclusion criterion for the statistical analysis of clinical data and immunohistochemical (IHC) staining of primary PCa for 139 patients was the availability of complete clinicopathological data, including prostate-specific antigen (PSA) follow-up data. The exclusion criteria for the patients included in the analysis of BCR-free survival time were neoadjuvant and adjuvant treatments. The analyses were performed using SPSS software, v22 (SPSS), IBM Corporation, Armonk, NY, USA. Mann–Whitney U and *t*-tests were used to compare differences between the two sample groups. Kruskal–Wallis and Dunn’s multiple comparison tests were applied to compare more than two groups. Wilcoxon matched-pairs signed-rank tests were used to compare paired samples (GS ≥ 4 + 3, PSA ≥ 10 ng/mL, pTNM ≥ T3a). Positive surgical margins (PSMs) were considered intermediate-to-high risk factors, and patient subgroups were formed according to these criteria. FGFRL1 staining patterns were categorized as either FGFRL1 low or FGFRL1 high, based on the mean IHC scores for the cell membrane, cytoplasmic, and nuclear FGFRL1 staining (37, 90, and 9, respectively), as described in the Supplementary Materials and Methods. The association between FGFRL1 expression and clinicopathological parameters was analyzed using an χ^2^ test; correlations between the FGFRL1 IHC and GSs, and the pre-operative PSA and Ki67 IHC scores, were determined using a Spearman’s correlation test. Kaplan–Meier survival analysis was conducted to study the time to biochemical recurrence (BCR), defined as two consecutive postoperative serum PSA values ≥ 0.2 ng/mL, with the first being the date of BCR. Comparison of two Kaplan–Meier curves was performed using a log-rank test. Cox PH univariate and multivariate regression analyses were used to determine the relationship between clinicopathological parameters and BCR-free survival time. A *p*-value ≤ 0.05 was considered statistically significant.

### 2.4. Cell Culture and Treatments

PC-3, LNCaP, VCaP, DU145, and NCl-H660 prostate cancer cell lines and benign PNT1a prostate epithelial cells were obtained from the American Type Culture Collection (ATCC) and the PC3M cell line was obtained from Caliper Life Sciences. PC-3 (RRID:CVCL_0035), PC3M (RRID:CVCL_9555), and DU145 (RRID:CVCL_0105) cells were cultured in DMEM (Gibco, Thermo Fisher, Paisley, Scotland, UK), containing 10% fetal bovine serum (FBS, Gibco, Thermo Fisher, Paisley, Scotland, UK ) and 1% antibiotics (Lonza, Verviers, Belgium). PNT1a (RRID:CVCL_2163), LNCaP (RRID:CVCL_0395), and VCaP (RRID:CVCL_2235) cells were cultured in RPMI medium (Gibco, Thermo Fisher, Paisley, Scotland, UK) with 10% FBS, 2 mM GlutaMAX (Gibco, Thermo-Fisher, Paisley, Scotland, UK ), and 1% antibiotics. NCI-H660 (RRID:CVCL_1576) prostate cancer cell line was cultured in RPMI medium with 5% FBS, 5.5 mg/mL transferrin (Sigma Aldrich, Saint Louis, MO, USA), 10 mg/mL insulin (Gibco, 12585-014, Paisley, UK), 10 nM hydrocortisone (Sigma Aldrich, Saint Louis, MO, USA), 10 ng/mL sodium selenite (Sigma Aldrich, Saint Louis, MO, USA) (40 nM), EGF (R&D systems, Minneapolis, MN, USA), 10 nM beta-estradiol (Sigma Aldrich, Saint Louis, MO, USA), 2 mM GlutaMax and 1% antibiotics. All cell cultures were maintained in a humidified incubator at 37 °C with 5% CO_2_. All human cell lines used in this study have been authenticated using short tandem repeat profiling within the last three years at IdentiCell Laboratories (Department of Molecular Medicine at Aarhus University Hospital Skejby, Århus, Denmark) and at the Institute of Molecular Medicine (FIMM), University of Helsinki, Finland. All experiments were performed with mycoplasma-free cells (Lonza LT07-118 mycoplasma detection kit). In cell signaling studies, cells were serum-starved in 0.1% bovine serum albumin (BSA, Sigma Aldrich, Saint Louis, MO, USA) for 24 h, and then treated with 25 ng/mL recombinant FGF8b (R&D systems, Minneapolis, MN, USA) or 25 ng/mL FGF2 (R&D systems, Minneapolis, MN, USA), along with 20 UI/mL heparin (Sigma Aldrich, Saint Louis, MO, USA). In addition, serum-starved PC3M cells were first treated with FGFR inhibitors BGJ398 or AZD4547, or MEK inhibitor PD98059 (Selleck Chemicals LLC, TX, USA) for 30 min; thereafter, FGF was added and cell lysates were harvested after 10 min.

### 2.5. FGFRL1 Knockdown and Overexpression in PC3M Cells

FGFRL1-shRNA plasmid (sc39967-SH) and control shRNA plasmid (sc108066) were purchased from Santa Cruz Biotechnology Inc., TX, USA. For the overexpression studies, a human full-length FGFRL1 sequence for amino acid residue 1–504 (plasmid #23600, Addgene Europe, Teddington, UK) was amplified using PCR and inserted into the EcoRI/BamH I site of the expression vector pEGFP-C2, and then tagged with EGFP. The construct was verified by restriction digestion and sequencing, transfected into PC3M cells using Lipofectamine™ 2000 (Thermo Fisher Scientific, Carlsbad, CA, USA), and stable transfected clones were selected using 6 µg/mL puromycin (Thermo Fisher, Paisley, Scotland, UK). Clones were examined for FGFRL1 protein knockdown (KD) or overexpression using Western blotting. This study used two PC3M control-KD clones (sh5 and sh8) and two PC3M FGFRL1-KD clones (sh9 and sh11). FGFRL1-KD in VCaP and LNCaP cells produced by siRNA and CRISPR-cas9 techniques failed, probably due to the essential nature of the target gene in these cells, resulting in poor cell viability.

### 2.6. Western Blotting

Western blot was used to detect protein expression levels in cell lysates and xenograft tissue lysates. The procedures and antibodies used are described in the Supplementary Materials and Methods. Uncropped Western blot images are presented in Figure S1.

### 2.7. RNA Isolation and Quantitative Real-Time PCR (qRT-PCR)

Total RNA was extracted from fresh frozen primary PCa and AdjPr tissue samples and cell cultures using an RNeasy Mini Kit (Qiagen, Hilden, Germany). Only patient tissue specimens with >50% carcinoma or benign glands, based on H&E staining, were used. Here, 1 µg of total RNA was transcribed into complementary DNA (cDNA) using Maxima reverse transcriptase (Thermo Fisher Scientific, Helsinki/Finland), RNA inhibitor (Promega, Fitchburg WI, USA), dNTP mix (Thermo Fisher Scientific, Helsinki/Finland), and Oligo dT primer (Oligomer Oy, Helsinki, Finland). qRT-PCR was performed using SYBR™ Green master mix (Thermo Scientific, Vilnius, Lithuania) with a Bio-Rad CFX96 Touch™ system, Bio-Rad Laboratories Oy, Helsinki, Finland. Primers for amplification of FGFRL1 (F: 5′-CCATGTGGACCAAGGATGGC-3′, R: 5′-CTAATGTCATCCAGCACGACG-3′); PTX3 (R: 5′-AGC ACT CGG AAT GGG ACA AG-3′, R: 5′-TAG CCG CCA GTT CAC CAT TT-3′); and FZD7 (R: 5′-GGT GCA GTG TTC TCC CGA A-3′, R: 5′-CAT GAA GTA GCA GCC CGA CA-3′) were designed based on the NCBI databases (http://www.ncbi.nlm.nih.gov/tools/primer-blast/, 17 February 2019). The threshold cycles were determined and the standard ΔΔCt method was used to calculate fold-change differences in mRNA expression. Expression levels of FGFRL1 were normalized to the TBP gene as a housekeeping standard (F: 5′-GAATATCCCAAGCGGTTT-3′, R: 5′-ACTTCACATCACAGCTCCCC-3′).

### 2.8. Cell Proliferation and Migration Assays

PC3M cells were seeded on a 96-well plate (5000 cells/well) in full-growth medium and imaged every 2 h with an Incucyte^®^ ZOOM imaging system (Essen BioScience, Royston Hertfordshire, UK) in an incubator at 37 °C and 5% CO_2_. Proliferation was monitored for four days, and kinetic image data were analyzed with Incucyte^®^ ZOOM software (Essen BioScience, Royston Hertfordshire, UK). Cell migration was examined using scratch wound assays and an Incucyte^®^ S3 imager device (Sartorius, Goettingen, Germany) according to the manufacturer’s instructions. Briefly, scratch wounds were generated in confluent cell layers on 96-well Imagelock plates with the “wound-maker” tool. Two images per well (10x objective, Sartorius, Goettingen, Germany) were collected every 2 h. The percentage of cell migration leading to wound confluence was analyzed with Incucyte^®^ S3 software (Sartorius Stedim Biotech, Goettingen, Germany). To examine the effects of the FGFR inhibitors BGJ398 and AZD4547 on cell proliferation or migration, the inhibitors were applied to cells one day after plating or at the time of wounding, respectively. The data were graphically presented using GraphPad Prism 8 software (GraphPad Software Inc., San Diego, CA, USA).

### 2.9. Organotypic 3D Culture

Organotypic 3D cell culturing was performed as described earlier [38]. Briefly, PC3M ctrl-KD and FGFRL1-KD clone 11 were seeded as single cells between two layers of Matrigel (Corning/BD Biosciences, Glendale, Arizona, USA) in the presence of 5% FBS-DMEM on 96-well angiogenesis µ-plates (Ibidi, Gräfelfing, Germany). The cells were allowed to form multicellular organoids for up to 12 days. FGFR inhibitors BGJ398 and AZD4547 were added on day four (1 µM, 5 µM, and 10 µM) and incubated for an additional eight days. The cultures were monitored using an Incucyte^®^ ZOOM real-time imaging device (Essen BioScience, Royston Hertfordshire, UK). Finally, organoids were stained with calcein AM and EthD1 dyes (Thermo Fisher Scientific, Carlsbad, CA, USA) to visualize living and dead cells, respectively, and imaged with a spinning disk confocal microscope (Zeiss Axiovert 200 M, Zeiss, Oberkochen, Germany, 5x objective). Images were converted to maximum-intensity projections with SlideBook6 software (3i Intelligent Imaging Innovations Inc., Denver, CO, USA) and analyzed using our in-house AMIDA software [39]. The resulting quantitative morphometric data were visualized and plotted using the R software environment (http://www.r-project.org, 16 October 2018).

### 2.10. Mouse Xenografts

Five-week-old male athymic nu/nu mice (Harlan Laboratories, Horst, The Netherlands) were used in the study. The mice (first experiment *n* = 12, second experiment *n* = 20) were randomized into two groups for subcutaneous inoculation with either control-KD (clone 8) or FGFRL1 KD-11 (clone 11) PC3M cells. Cells were collected at the log growth phase and subcutaneously injected into mice (1 × 10^6^ cells in 100 µL of PBS). Mice were housed under controlled conditions (12 h light/12 h dark cycles, temperature 21 ± 3 °C) and fed standard chew food and tap water ad libitum. Tumors were grown for 45 days, and the tumor sizes and body weights of mice were measured every five days. After sacrificing the mice with CO_2_ asphyxiation followed by cervical dislocation, tumors were excised and divided into two parts: one for fixation with 10% neutral-buffered formalin, and another for protein and RNA expression analysis. Xenograft tissues were analyzed with H&E staining and immunostaining to examine key processes in cancer tissues (see Supplementary Materials and Methods ).

### 2.11. RNA Sequencing and Data Analysis

Total RNA was harvested from PC3M ctrl-KD cells (clones 5 and 8, each in two replicates) and PC3M FGFRL1-KD cells (clone 11, two replicates) and from xenograft tumors (ctrl-KD, *n* = 5; FGFRL1-KD-11, *n* = 5) as described above. Differentially expressed gene sets (FGFRL1-KD vs. ctrl-KD) were further analyzed by bioinformatic procedures described in the Supplementary Materials and Methods. The sequencing coverage and quality statistics for each sample are summarized in Table S6.

## 3. Results

### 3.1. FGFRL1 mRNA Expression Is Increased in PCa

Analysis of public genomic databases suggested a significant upregulation of the expression of FGFRL1 mRNA in PCa, compared to normal prostate samples (Figure S2A). A more detailed analysis of FGFRL1 gene expression in the large MSKCC microarray gene expression data set (218 prostate cancers) [32] showed that FGFRL1 mRNA levels were significantly increased in primary PCa compared to normal prostate, and further increased in advanced and metastatic PCa (Figure S2B). The expression of FGFRL1 was higher in Gleason 9 and stage 4 tumors compared to cases with lower Gleason scores and stages. Its expression correlated with lymph node metastasis (Figure S2B, *** *p* < 0.001). 

Prompted by the MSKCC gene expression microarray data, we investigated the expression of FGFRL1 mRNA in primary PCa samples from RPs of 48 PCa patients. We used quantitative qRT-PCR to measure FGFRL1 mRNA expression in tissue samples of primary PCa (*n* = 48) and adjacent non-malignant prostate (AdjPr) available from 36 RPs of the patients. The results demonstrated that the relative FGFRL1 mRNA levels were upregulated in PCa tissues compared to AdjPr tissues (Figure S2C, *p* < 0.0001), in line with the MSKCC data [32].

### 3.2. FGFRL1 Protein Shows Increased Expression and Altered Cellular Localization in HGPIN and PCa Compared to Benign Prostate

**Figure 1:**
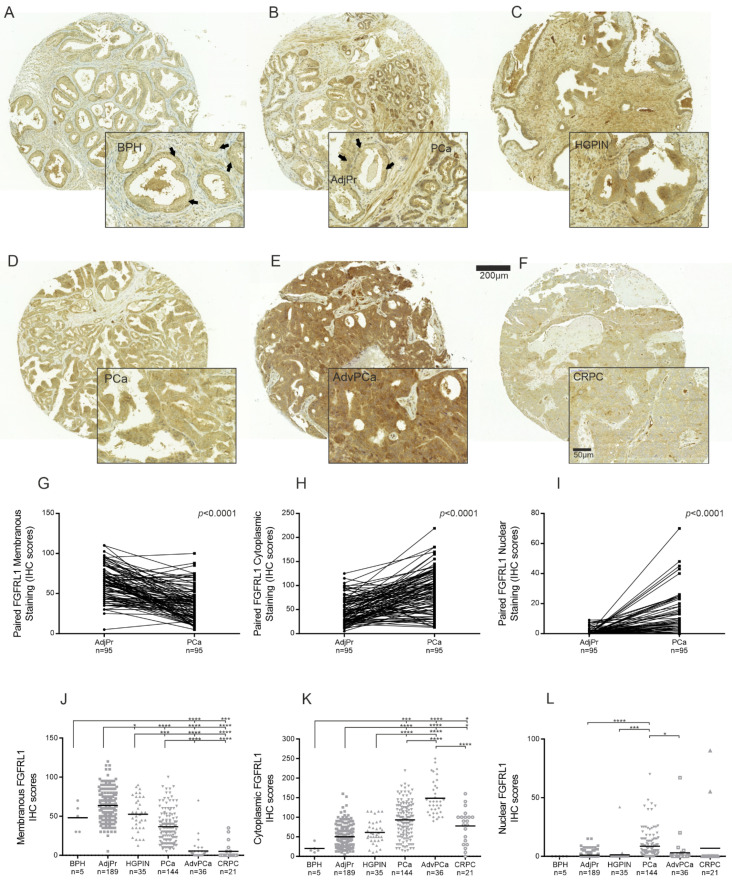
Immunohistochemical (IHC) staining of FGFRL1 in benign prostate and PCa tissue. (**A**–**F**), Representative FGFRL1 IHC staining is shown in benign prostate hyperplasia (BPH) (**A**), adjacent benign prostate tissue (AdjPr) (**B**), high-grade PIN (HGPIN) (**C**), primary PCa (**B**,**D**), advanced PCa (AdvPCa) (**E**), and castration-resistant PCa (CRPC) (**F**). The arrows point to representative areas with clear membranous FGFRL1 staining (**A**,**B**, inserts). (**G**–**I**), Comparison of cell membrane (**G**), cytoplasmic (**H**), and nuclear (**I**) FGFRL1 IHC scores in paired specimens in TMA I. (**J**–**L**), Comparison of cell membrane (**J**), cytoplasmic (**K**) and nuclear (**L**), FGFRL1 IHC scores in BPH, AdjPr, HGPIN, PCa, AdvPCa, and CRPC. Black bars represent the mean IHC score of each group. The difference among groups was analyzed by Kruskal–Wallis test and Dunn’s multiple comparison test. n is patient number. * *p* < 0.05, ** *p* < 0.01, *** *p* < 0.001, **** *p* < 0.0001.

Next, we studied whether increased FGFRL1 mRNA gene expression resulted in altered protein levels. We immune-stained TMAs containing samples from BPH, AdjPr, HGPIN, and primary PCa (TMA I), AdvPCa (TMA II), and CRPC (TMA III) for the FGFRL1 protein. In non-malignant tissues (BPH and AdjPr), luminal epithelial cells showed clear membrane-associated FGFRL1 staining, often in combination with weak cytoplasmic staining (Figure 1A,B). In HGPIN and primary PCa, membranous staining was less clear, but increased cytoplasmic and positive nuclear staining were often observed (Figure 1C,D). In both AdvPCa and CRPC, IHC staining of FGFRL1 was primarily cytoplasmic (Figure 1E,F). Human liver and colon cores served as positive controls in TMAs (Figure S3). Stromal cells consistently showed weak to moderate FGFRL1 staining. The IHC scores were used to quantify and compare FGFRL1 staining across the entire panel of luminal epithelial and cancer cells [35]. In pairwise analyses of AdjPr and PCa from the same patients in TMA I, membrane-associated staining was significantly weaker in PCa (Figure 1G), while IHC scores for cytoplasmic and nuclear FGFRL1 staining were significantly higher in cancer tissues compared to AdjPr (Figure 1H,I, respectively). IHC scores further showed a gradual decrease in membranous FGFRL1 staining from AdjPr to AdvPCa and CRPC (Figure 1J), while cytoplasmic FGFRL1 showed a reciprocal, stepwise increase (Figure 1K). Nuclear FGFRL1 levels were higher in primary PCa tissues than in AdvPCa and CRPC (Figure 1L). This tendency did not reach statistical significance for CRPC, probably due to the small number of samples included (*n* = 21). Nuclear FGFRL1 was also detected in some AdjPr samples. None of the BPH and only a few HGPIN specimens showed nuclear FGFRL1 staining.

### 3.3. Expression of FGFRL1 Correlates with Gleason Score, Pre-Operative PSA Levels, and Ki67 Staining

**Figure 2:**
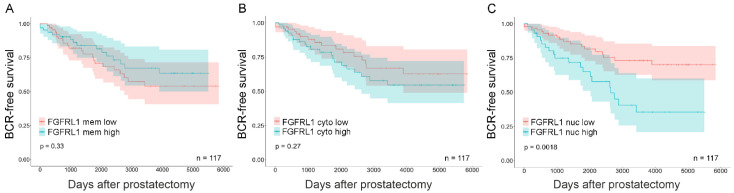
Kaplan–Meier analysis for membrane, cytoplasmic, and nuclear FGFRL1 expression associated with BCR-free patient survival in primary PCa. Primary PCa cases were stratified based on localized FGFRL1 IHC score. (**A**) low cell membrane (FGFRL1 mem low, red) versus high cell membrane (FGFRL1 mem high, blue), (**B**) low cytoplasmic (FGFRL1 cyto low, red) versus high cytoplasmic (FGFRL1 cyto high, blue), and (**C**) low nuclear (FGFRL1 nuc low, red) versus high nuclear (FGFRL1 nuc high, blue). 95% confidence interval is shown. *n* = 117, no neoadjuvant or adjuvant treatment.

Complete clinical follow-up information was available for 139 (97%) patients of 144 primary PCa patients (TMA I). Median follow-up time was 46 (inter-quartal range, 24–114) months. The samples were stratified based on their localized FGFRL1 IHC scores (high vs. low) and association with age, Gleason score (GS), PSA-level, pT-status, and PSM. Significant associations were found between the GS (≤7 vs. 8–9) and membranous and nuclear staining (Table S1). Membranous staining showed a significant negative correlation with GS (*r* = −0.200, *p* = 0.018) and Ki67 IHC staining (*r* = −0269, *p* = 0.002) (Table 1). Similarly, cytoplasmic FGFRL1 localization displayed a significant positive correlation with GS (*r* = 0.217, *p* = 0.010) and Ki67 IHC staining (0.261, *p* = 0.002) (Table 1). The strongest correlation was observed between nuclear staining and GS (*p* < 0.0001). Nuclear staining also correlated with pre-operative PSA (*p* = 0.019). Low membranous and high cytoplasmic FGFRL1 levels further showed a tendency to indicate reduced disease-free lifetime after radical prostatectomy (Figure 2A,B, respectively). In contrast, nuclear staining was positively correlated with GS (*r* = 0.297, *p* < 0.0001) and pre-operative PSA (*r* = 0.199, *p* = 0.002) (Table 1) and associated with PSM (Table S1). Only men in the high nuclear FGFRL1 group showed a significantly shorter time to BCR (Figure 2C). High nuclear FGFRL1 was an independent prognostic marker for a shortened BCR-free survival time after RP (HR 3.535; 95% CI 1.747-7.153, *p* < 0.0001, Table S2).

### 3.4. FGFRL1 Negatively Regulates FGFR Signaling in Prostate Cancer Cells

Next, the expression and function of FGFRL1 was studied in experimental models. The FGFRL1 protein was detected at low levels in non-malignant PNT1a prostate epithelial cells and at higher levels in several PCa cell lines (Figure S4A). For functional studies, PC3M, LNCaP, and VCaP cells were subjected to FGFRL1 silencing experiments using shRNA vectors. Additional CRISPR-cas9 KD experiments with LNCaP and VCaP failed due to poor survival of the FGFRL1 knockdown cells (not shown). Knockdown clones with decreased FGFRL1 levels were validated by qRT-PCR and Western blotting (Figure S4B,C). PC3M clone 11 exhibited approximately 75% stable knockdown at RNA and protein levels and was used for further experiments, in contrast to 50% for clone 9 (Figure S4B,C). Finally, stable functional overexpression of FGFRL1 was achieved by the transient transfection of PC3M cells with a pEGFP-tagged FGFRL1 plasmid, as verified by Western blotting (Figure S4D). Ctrl-KD PC3M cells showed clear endogenous FGFRL1 staining at the cell membranes, but the membranous protein was not detectable in FGFRL1-KD PC3M cells (Figure S4E). Strong immunostaining of FGFRL1 was detected in a subpopulation of cells transfected with pEGFP-FGFRL1 (Figure S4F).

**Figure 3:**
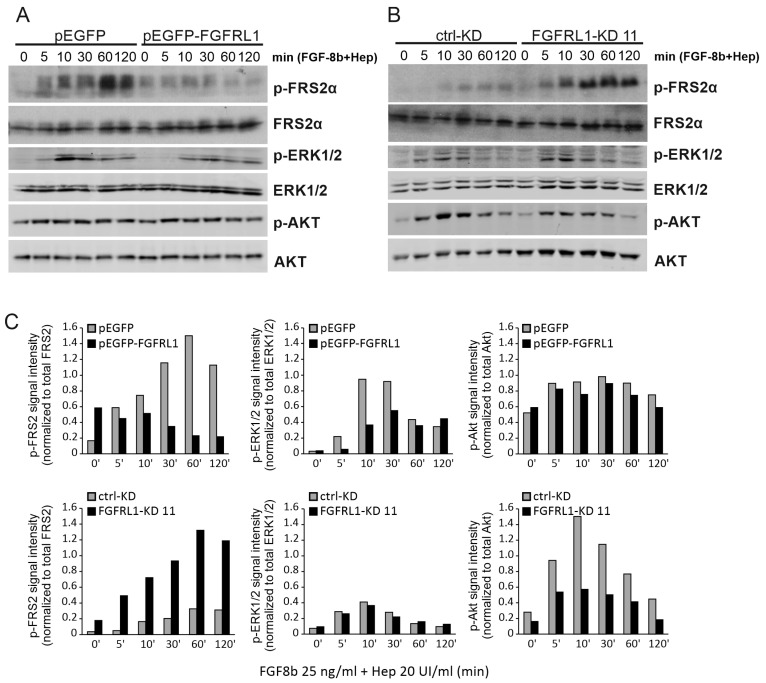
Western blot analysis of FGF-induced signaling in FGFRL1-modified tumor cells. (**A**) PC3M cells transfected with FGFRL1 overexpression (pEGFP-FGFRL1) or control vector (pEGFP). (**B**) sh-RNA transfected PC3M cells with stable FGFRL1 knockdown (FGFRL1-KD 11) and scrambled control (ctrl-KD). In both cases, serum-starved cells were treated with FGF-8b (25 ng/mL) and heparin (20 UI/mL) for indicated times. (**C**) Quantification of signal intensities of p-FRS2α, p-ERK1/2, and p-Akt (Ser473) in Western blot. The phospho-protein intensity values were obtained using Image J and normalized against the corresponding total protein intensity in a given sample.

PC3M cells with FGFRL1 knockdown or overexpression were used to investigate the phosphorylation of downstream FGFR targets upon stimulation with the ligand FGF8b. The Western blot of FGFRL1 expression in these cell lines and their controls is presented in Figure S4C,D. FGFRL1 has a high binding ability to FGF8b [19]. Overexpression of FGFRL1 effectively attenuated the phosphorylation of FGFR-substrate 2α (FRS2α), which is typically activated by FGFs including FGF8b (Figure 3A,C, upper panel) [6]. In contrast, FGF8b evoked a more robust FRS2α phosphorylation response in FGFRL1-KD cells compared to the control (Figure 3B,C, lower panel; see also Figure S5), suggesting a decoy functionality for FGFRL1. Furthermore, analysis of the Ras/Raf/MAPK signaling cascade induced by FGF8b showed that phosphorylation of ERK1/2 was delayed and less pronounced in FGFRL1-overexpressing cells compared to controls (Figure 3A,C, upper panel). However, phosphorylation of ERK1/2 in FGFRL1 knockdown cells upon FGF8b stimulation was not altered compared to controls (Figure 3B,C, lower panel). PI3K/AKT signaling was unchanged in FGFRL1-overexpressing cells (Figure 3A,C, upper panel), while phosphorylated AKT showed a weaker signal in FGFRL1-KD cells stimulated by FGF8b (Figure 3B,C). Corresponding results were obtained with FGF2 treatment, which binds FGFRL1 with less affinity than FGF8b [19] (data not shown). 

### 3.5. FGFRL1 Knockdown Affects PC3M Organoid Growth and Differentiation in Organotypic 3D Cultures

**Figure 4:**
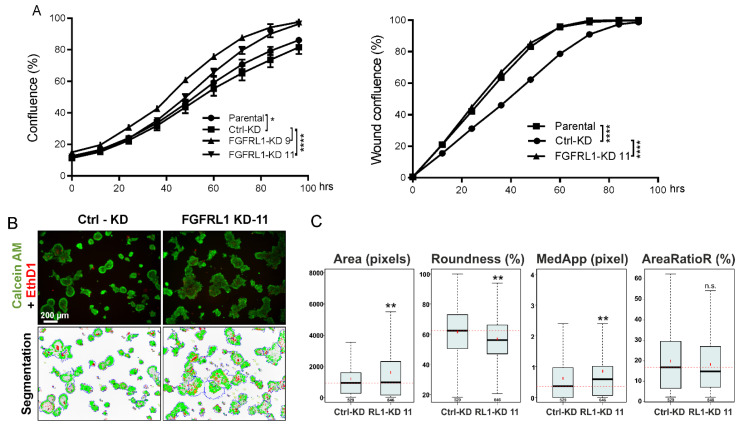
Functional analysis of PC3M cells with stable knockdown of FGFRL1 in vitro. (**A**) Cell proliferation (left) and migration (right) of control (ctrl-KD) and FGFRL1 knockdown (FGFRL1-KD) PC3M cells in 2D cultures were examined using IncuCyte imaging device in real time (two-way ANOVA, * *p* < 0.05, **** *p* < 0.001). (**B**) Cells were cultured in organotypic 3D culture system in Matrigel for 12 days. At the end point, the cells were stained with Calcein AM and Ethidium homodimer (EthD-1) and imaged with confocal microscope (upper panel). Phenotypic analysis was performed with AMIDA software (lower panel, image segmentation). (**C**) Quantitation of object size (*Area*), the shape of objects (*Roundness* and *MedApp*), and the relative amount of red fluorescence in an object (*AreaRatioR*), indicating cell death, are presented as box-and-whisker plots (Bonferroni-corrected *t*-test, ** *p* < 0.001; n.s., not significant). The total number of analyzed objects (in 6 replicate wells) is indicated under the whisker of the box-and-whisker plots.

The effect of FGFRL1 on PCa cell growth and migration of parental PC3M, control-KD, and stable FGFRL1-KD clones 9 and 11 was investigated in 2D monolayer cultures by monitoring confluence (%) and wound healing in vitro. FGFRL1-KD cells showed only slightly increased cell growth compared to parental and control-KD cells over four days of culturing (Figure 4A, left panel). The cell migration of FGFRL1-KD cells was slightly enhanced compared to control-KD cells (Figure 4A, right panel).

To examine the role of FGFRL1 in PCa cells under conditions that mimicked the tumor microenvironment in vitro, PC3M control-KD and FGFRL1-KD cells were cultured in an organotypic 3D cell culture system, supporting physiologically relevant cell–cell and cell–matrix interactions and promoting epithelial differentiation. Most organoids formed by control-KD cells matured to round, well-differentiated, and polarized structures with clear boundaries, strong cell–cell adhesion, and a detectable lumen—hallmarks of PC3M and PC3 cells in Matrigel (Figure 4B). By contrast, the polarization, shape, and symmetry were significantly disturbed in organoids formed by FGFRL1-KD cells (Figure 4B), which also lacked a hollow lumen. The average size of FGFRL1-KD spheroids was slightly but significantly increased, according to quantitative image analysis (Figure 4C, *Area*). The increasingly irregular shape of FGFRL1-KD organoids was characterized by reduced roundness and increased numbers of protrusions in the organoids (Figure 4C, *Roundness* and *MedApp*). Cell death was not significantly affected in the FGFRL1-KD spheroids (*AreaRatioR*).

Organoids were also treated with the FGFR inhibitors BGJ398 and AZD4547, starting from day four. FGFRL1-KD organoids showed significantly reduced sensitivity to BGJ398 in 3D cultures at 5 µM concentration (Figure S5A,B). Additionally, cell death was more pronounced in control-KD cells treated with 5 µM of BGJ398 than in FGFRL1-KD cells (Figure S5B, *AreaRatioR*). Statistically significant differences in responses to AZD4547 were also observed at 1 µM concentration (Figure S5B, *Area* and *AreaRatioR*). No significant differences were observed in 2D monolayer cultures (Figure S5C). The presence and functional activity of FGFRL1 may thus sensitize organoids specifically to the effects of FGF inhibitors, while its absence protects against these inhibitors. Our observations were in line with the presumed function of FGFRL1 as a decoy receptor that reduces FGF signaling and FGF-induced epithelial maturation in host cells, which may be more critical for cell growth and survival under organotypic 3D growth conditions in a laminin-rich matrix that strongly promotes cell differentiation. The functionality of the FGFR inhibitors in inhibiting FGF-induced signaling in these cells was verified by Western blotting (Figure S5D). The MEK inhibitor PD98059 was used as a negative control for FGFR-specific FRS2α phosphorylation. 

### 3.6. Growth of Xenografts Derived from FGFRL1-KD Cells Is Decreased

**Figure 5:**
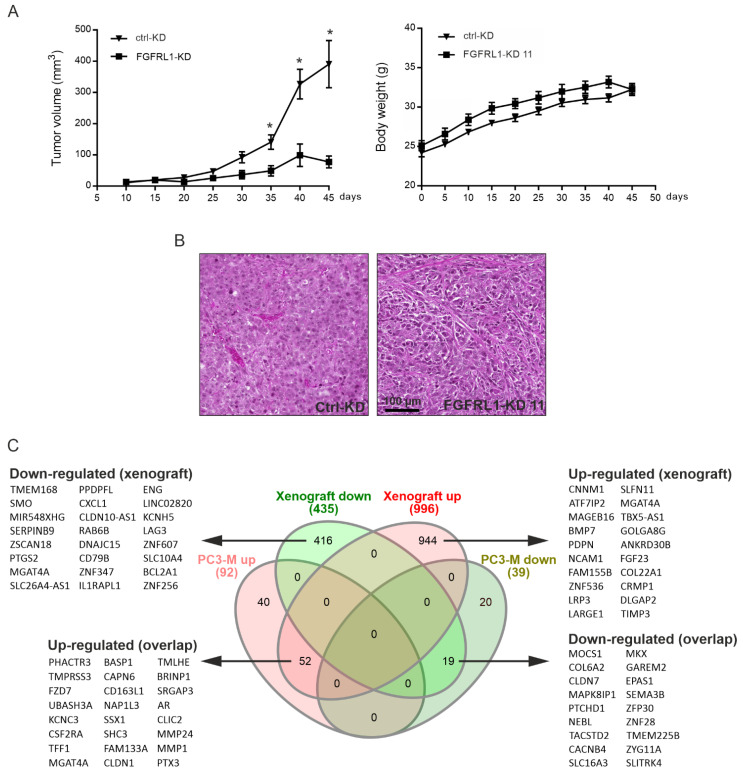
Growth and gene expression analysis of PC3M FGFRL1-KD and control-KD xenografts. (**A**) Growth of the cells as subcutaneous xenografts and body weight of mice bearing the tumors (* *p* < 0.05, student’s t-test for each time point, *n* = 10 for each group). (**B**) HE-stained histological sections of a representative xenograft of control-KD (ctrl-KD) and FGFRL1-KD (FGFRL1-KD11) groups. (**C**) Analysis of differential gene expression after shRNA-mediated FGFRL1 knockdown in PC3M cell line and mouse tumor xenografts generated from PC3M cells, in comparison to corresponding control cells/tumors. Venn diagrams show genes altered with a fold change > 2-fold with FDR of < 0.001. Core genes in xenograft data and overlapping between cell line and xenograft data are listed beside the diagrams.

The role of FGFRL1 in regulating PCa cell growth in vivo was studied by inoculating the stable PC3M clone FGFRL1-KD 11 and control-KD cells subcutaneously into athymic nude male mice. All mice in the control group generated tumors (*n* = 16), but tumors grew in only 13 of the 16 mice in the FGFRL1-KD group. Consistently reduced FGFRL1-KD expression in tumors was validated by qRT-PCR after 45 days (data not shown) and by decreased overall immunohistochemical staining (Figure S6). FGFRL1-KD tumors grew at a significantly slower rate and produced smaller xenografts, while the body weights of mice did not differ (Figure 5A); however, IHC staining of the KD tumors for PHH3 did not differ significantly from that of control tumors (*p* = 0.6580, Figure S7A). The H&E-stained sections of FGFRL1-KD tumors also showed a denser and seemingly less angiogenic histology than the control-KD tumors (Figure 5B), but IHC staining for CD34 did not show a statistically significant difference (*p* = 0.2028, Figure S7B). Nevertheless, the cell-cycle-related proteins cyclin E, cyclin-dependent kinase 2 (CDK2), and CDK4 were expressed at significantly lower levels in FGFRL1-KD tumors (Figure S7C,E–G), in line with the reduced growth of xenografts. By contrast, cyclin D1 and CDK6 showed no significant differences (Figure S7C,D,H).

### 3.7. Altered Gene Expression Profiles between Control-KD and FGFRL1-KD Cells in Cultured Cells and Xenografts

To investigate the molecular mechanisms of FGFRL1 in regulating PCa, RNAs from control-KD and FGFRL1-KD PC3M cells were sequenced. The analysis of the expression data resulted in 131 differentially expressed genes with a logarithmic fold change (log2FC) ≥ 1 or ≤ −1) corresponding to at least a two-fold change of expression (93 upregulated, 38 downregulated) and false discovery rates (FDRs) ≤ 0.05 (Figure 5C, Venn diagram). In parallel, we sequenced the mRNA from five FGFRL1-KD xenografts and five matching control tumors. RNA-seq data from xenograft tumors indicated a total of 1431 genes, differentially expressed in FGFRL1 KD xenografts compared to controls, with log2-fold changes (log2FCs) ≥ 1.5 and ≤ −1.5, and FDRs ≤ 0.01. Of these genes, 997 were upregulated and 434 were downregulated (Figure 5C). The Spearman correlation between the RNA-seq values of cell lines and xenografts was +0.87, indicating overall good consistency between the in vitro and in vivo models. A total of 71 genes (52 upregulated, 19 downregulated) were differentially expressed in both the PC3M FGFRL1-KD cells and xenografts, with comparable fold changes, the most significant of which are listed in Figure 5C. The top 71 unidirectionally up- and downregulated genes in both cell lines and xenografts are shown in Table S3A,B, respectively. They included upregulated genes for Frizzled Class Receptor 7 (*FZD7*), pentraxin-3 (*PTX3*), protease-like matrix metalloproteinase 1 (*MMP1*) and 24 (*MMP24*), or cathepsin B (*CTSB*)—all active in ECM turnover—and downregulated genes such as collagens (*COL6A2*) and semaphorin 3B (*SEMA3B*). Differential expression of *PTX3* and *FZD7* in FGFRL1-KD cells and xenograft tumors was validated by qRT-PCR in FGFRL1-KD xenografts (Figure S8).

The top 100 differentially expressed up- and downregulated genes in FGFRL1-KD xenografts compared to control tumors are listed in Table S4A,B, respectively, and partially in Figure 5C. The top upregulated genes in xenografts included those for the bone morphogenic protein 7 (*BMP7*), cell adhesion protein *NCAM1*, and protease inhibitor *TIMP3*, which modulate tumor–host interactions and may suppress tumor growth. The top downregulated genes included, for example, the signal transducers smoothened (*SMO*), *SERPINB9* and *CXCL1*.

**Figure 6:**
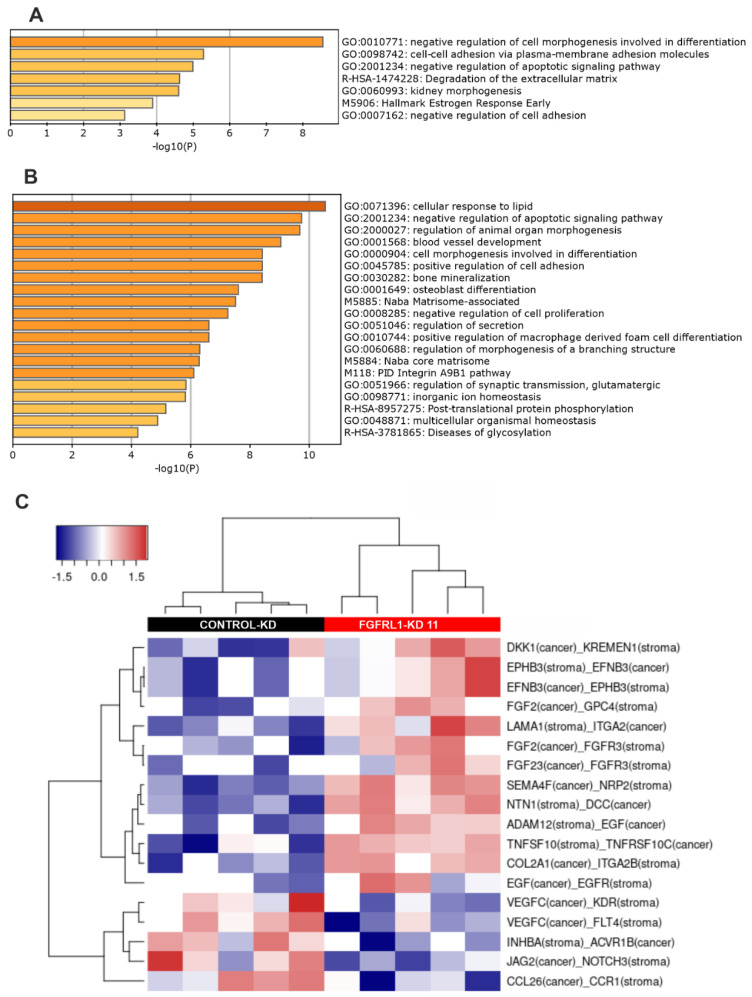
Bioinformatic analyses of differentially expressed (DE) genes in PC3M cell line and mouse xenografts. (**A**) Significantly enriched gene ontology (GO) categories and pathways in 71 DE genes identified in both PC3M cells and the mouse xenograft tumors, after FGFRL1 knockdown. Genes were analyzed by gene set enrichment analyses (GSEA) in the MSigDb database (http://www.gsea-msigdb.org, 10 January 2021) and visualized using Metascape (www.metascape.org, 15 January 2021). (**B**) Enriched GO categories and pathways in DE gene set (100 genes up and 100 genes down) identified in FGFRL1-KD xenograft tumors using GSEA and Metascape for visualization. (**C**) Heat map indicating significantly enriched tumor–stroma interactions detected by the CASTIN algorithm in FGFRL1-KD xenografts compared to controls [40]. Stroma: corresponds to reads matched to the mouse genome (=mouse stromal cells in xenografts). Tumor: corresponds to reads matched to human genome (=PC3M cells in xenografts).

GO (and GSE) analyses for the 71 overlapping genes in both FGFRL1-KD cell lines and xenografts resulted in seven significantly enriched GO categories and gene sets with *p*-values ≤ 0.001 (Figure 6A). The most significant GO/gene set categories were related to cell and tissue morphogenesis, apoptosis, cell adhesion, and ECM turnover. The GSE and GO analyses for the 100 most prominently altered up- and downregulated genes affected by FGFRL1-KD in xenografts (Table S4A,B) revealed highly overlapping, but more significant, GO categories, again related to cell and tissue morphogenesis and the regulation of apoptosis, but also to the negative regulation of proliferation, adhesion, angiogenesis, the matrisome composed of ECM proteins, and ECM turnover (Figure 6B). These, and more specific pathways such as bone mineralization and osteoblast differentiation, are highly characteristic of FGF/FGFR regulation [4,5,8]. Simultaneously affected pathways and mechanisms may explain the reduced growth of FGFRL1-KD xenografts. 

### 3.8. Altered Ligand–Receptor Interactions in Xenografts

Because FGFRL1 xenograft growth was strongly inhibited, the CASTIN analysis tool [40] was used to identify significantly enriched tumor–stroma interactions between receptors and cognate ligands, expressed specifically by mouse cells (stromal cells) and human tumor cells (PC3M cells). The heat map (Figure 6C and Table S5) indicates 18 interactions enriched in FGFRL1-KD xenografts, including growth and differentiation factors with their respective ligands, including the pairs SEMA4F–NRP2, EFNB3–EPHB3, FGF2–FGFR3 or GPC4, EGF–EGFR, VEGFC–KDR or FLT4, and INHBA–ACVR1B3. Additional connections suggest the inhibition of Wnt signaling (DKK1–KREMEN) and mechanisms with tumor suppression capacity (DCC–NTN1). A particularly interesting potential interaction with high relevance for tumor biology includes *JAG2* (cancer) with NOTCH3 (mouse stroma), and interactions of integrins (ITGA2, ITGA2B) with ECM proteins (COL2A1, LAMA1). Most of these represent altered tumor–host cell interactions, in line with the GO and GSEA analyses, which may functionally relate to the observed differences in tumor proliferation and morphology. 

## 4. Discussion

We demonstrate for the first time that increased FGFRL1 expression may play a role in PCa progression in a significant proportion of PCa patients. Our results also show the re-localization of membranous FGFRL1 in nonmalignant prostate to cytoplasmic and nuclear sites in PCa. Importantly, decreased membranous and increased intracellular FGFRL1 levels correlated with adverse clinicopathological features of primary PCa, and the occurrence of nuclear staining was associated with a shortened time to BCR in PCa patients. 

The potential routes and mechanisms of the diverse intracellular localizations of FGFRL1 are not clear. The newly synthesized FGFRL1 is first transported to the cell membrane, and the protein may then be internalized by endosomes for recycling or degradation. The short intracellular domain of FGFRL1 may affect the turnover and functions of FGFRL1 [17,41]. The extracellular domain of FGFRL1 can also be cleaved in the proximal region and shed from the cell surface in a soluble form [19]. Our observation of altered subcellular localization could indicate that newly synthesized FGFRL1 in PCa cells is not properly transported to the cell membrane but, instead, accumulates in the cytoplasmic organelles. Altered internalization and defective trafficking of membranous FGFRL1 in tumor cells could also lead to the observed localization of FGFRL1 in nuclei or cytoplasm [17,19,42].

There is compelling evidence for the nuclear localization and functioning of other FGFRs in FGFR-related cancers and congenital diseases [42,43,44,45], but the nuclear localization of FGFRL1 and its potential functional consequences have not been previously identified. Our results suggest that decreased membranous FGFRL1 staining, concomitant with increased cytoplasmic and nuclear FGFRL1, is associated with aggressive PCa. Specifically, a shortened time to BCR can be expected for men with high nuclear staining in primary PCa. This adverse prognostic marker could be considered in patient surveillance. Nuclear staining of FGFR1 has also been reported in breast cancer [44] and pancreatic cancer, where this pattern has been associated with poor prognoses [43]. The mechanisms of nuclear FGFRL1 remain to be studied. Our clinical results suggest, however, that they could be associated with prostate cancer evolution. Based on the results of cellular trafficking of other FGFRs, it is possible that either the full-length FGFRL1 protein or the cleaved intracellular domain can be transported to the nucleus [42]. However, our immunohistochemical analyses of clinical specimens with a polyclonal anti-FGFRL1 antibody could not differentiate between various domains of the protein. An interesting possibility is that FGFRL1 could also in the nucleus act via FGFR1 [21], which has been reported to have nuclear functions in breast cancer [44].

The extracellular domain of FGFRL1 is strongly homologous with FGFR1-4 and effectively binds at least FGF8 and FGF2 ligands. Both membrane-bound and soluble FGFRL1 are capable of sequestering FGFs with high affinity and thus acting as decoy receptors for FGF signaling [19,21]. Our experiments with FGFRL1-KD PC3M cells treated with FGF2 and FGF8b, which both bind to FGFRL1 [11,12,46], showed more robust phosphorylation of FRS2α compared to control cells. By contrast, the phosphorylation of FRS2α decreased when FGFRL1 was overexpressed, and this was also accompanied by decreased downstream phosphorylation of ERK1/2. These results supported the suggested decoy receptor function of membranous FGFRL1 in cancer cells such as PC3M retaining at least partial membranous FGFRL1. Particularly in the nonmalignant prostate epithelium with almost exclusive membranous localization of FGFRL1, decoy function of FGFRL1 could be prevalent. Indeed, it is possible that, in PCa, due to the loss of membranous FGFRL1, re-localized FGFRL1 fails to regulate FGFR signaling through the decoy receptor function and/or adopts other functions to promote PCa progression, as happens, for example, in ovarian cancer [27].

A limitation of the current study is that efficient and stable FGFRL1 knockdown without compromising cell viability was achieved only in PC3M cells, and thus only these cells could be used in experiments that required a lasting, stable level of knockdown, such as mouse xenografts. Parallel experiments with LNCAP and VCAP cells, using both shRNA and CRISPR-cas-9-mediated methods, did not result in viable cell clones. PC-3 cells are a commonly used prostate cancer cell line, which represent an aggressive, castration-resistant, and androgen-independent form of PCa. In PC3M cells, several knockdown clones were generated and we used the clone (sh11) with the most prominent and sustained knockdown for most experiments. Our in vitro experiments showed that KD of FGFRL1 did not have strong effects on the proliferation or migration of PC3M cells in cultures; however, reduced FGFRL1 expression modified the differentiation potential of PC3M cells, especially when embedded in laminin-rich Matrigel. This was indicated by characteristic organoid morphologies, such as reduced roundness, increased irregularity, and more pronounced invasive properties in 3D cultures. This was in line with the hypothesis that FGFRL1 acts as a decoy receptor under these conditions. Loss of the normal functions of FGFRL1could be linked to an increase in pro-tumorigenic FGF signaling, resulting in the simultaneous loss of differentiation-related aspects such as cell–cell contact, polarization, and the formation of a basement membrane in 3D cultures [38,39]. Reduced FGFRL1 expression further lowered the sensitivity of PC3M organoids to small-molecule FGFR inhibitors, possibly resulting from uninhibited growth-stimulatory FGFR pathway activities. These in vitro results could reflect the consequences of the loss of membranous FGFRL1 functions and adoption of other putative intracellular functions in malignant prostate tumors.

When FGFRL1-KD PC3M cells were implanted into nude mice, the tumor take rate markedly decreased, and the formation of xenografts was considerably slower than that of control cells. This result was somewhat surprising in light of our in vitro results; however, the decreased in vivo growth of FGFRL1-KD xenografts could be expected considering the clinical data that indicated increased FGFRL1 in association with tumor growth and progression. Besides having a membrane localization-associated decoy function, which could primarily function in normal cells and tissues, FGFRL1 may, in a tumor tissue context, contribute to PCa progression by other modes of action, such as an FGF-binding-dependent co-receptor function via FGFR1 [21]. At the time of harvesting the xenograft tumors for histological and molecular examination, the levels of cell-cycle-related proteins cyclin E, CDK2, and CDK4 were significantly decreased in FGFRL1-KD tumors, although the expression of PHH3 or the level of cleaved caspase 3 (not shown) did not significantly differ between KD and ctrl-KD xenografts. Compared to control-KD tumors, FGFRL1-KD tumors showed a tendency towards decreased CD34 immunostaining of capillaries, which may have contributed to the decreased growth of FGFRL1-KD tumors. Bioinformatics analyses of differential mRNA expression also suggested that pathways related to angiogenesis and blood vessel formation may be affected by FGFRL1-KD; however, the results for the in vivo and in vitro models indicated that FGFRL1 may not have pronounced direct effects on cell proliferation and cell cycle progression. FGFRL1 knockout in ESCCs similarly decreased xenograft growth without greatly affecting proliferation [28,29]. Other mechanisms, such as altered interactions with the tumor microenvironment (ECM, stromal cells, cell adhesion, integrin signaling, etc.), only partially present in the in vitro settings, may therefore be responsible for the observed changes in xenograft growth in vivo. A decrease in the negative-action decoy receptor FGFRL1 could also allow repressive stromal-derived FGFR signaling [5,10,11]. By analogy, increased and relocalized FGFRL1 expression, as observed in many clinical PCa tissue samples, may contribute to growth-promoting signaling.

RNA sequencing of PC3M FGFRL1-KD and control cells, and resulting mouse xenografts, indicated significant enrichment of the same cellular pathways and mechanisms. The xenografts generally showed more pronounced differences in gene expression (>1500 genes significantly changed) than the cell lines in the 2D culture (>120 genes). Nevertheless, the correlation (ρ = +0.87) between the two experiments was high, and over 70 genes showed similar expression patterns, with comparable fold changes and identical directions of change. GSE and GO analyses of the genes altered in FGFRL1-reduced xenografts and cell lines showed overlapping significant changes in functional pathways that affected cell and tissue morphogenesis, differentiation, and organ development, specifically in epithelial tissues. However, downregulated genes were mainly related to the negative control of apoptosis and the turnover of the ECM. GSE and GO analyses of the xenografts alone also indicated the enrichment of differentially expressed genes involved in the inhibition of proliferation and the regulation of cell adhesion, angiogenesis, bone formation, and the matrisome in FGFRL1-KD tumors. These results pointed toward a primary role of FGFRL1 and associated gene signatures in the modulation of differentiation processes and, under in vivo conditions, in the regulation of tumor–stromal interactions. These processes could relate to the observed changes in xenograft growth and the clinical progression of PCa; however, validation of these assumptions requires further experimental investigation.

To explain our experimental and clinical observations, we focused first on differentially expressed genes observed in both FGFRL1-KD xenografts and cell cultures. These included *PHACTR3*,*PTX3*, *TMPRSS3*, and the Wnt receptor *FZD7*, for which functional associations with FGFRL1 or FGFR signaling have not been previously reported. The results for *PTX3* and *FZD7* were also validated in the xenografts. Interestingly, the *PTX3* gene, upregulated in both FGFRL1-KD xenografts and cell lines, has been shown to inhibit the angiogenic activity of FGF2 [47]. PTX3 is a pattern-recognition receptor that acts as an FGF antagonist, blocking the growth and vascularization of FGF-regulated tumor types, including PCa [48,49]. FGFRL1-KD, via increasing PTX3, could thus strongly affect cellular functions, tumor growth, and vascularization. PTX3 also has suppressive effects on the progression of bladder cancer [49].

The comparison of differentially expressed genes, specifically in FGFRL1-KD xenografts versus controls, revealed high-ranking upregulated genes such as *BMP7* [50] and *TIMP3* [51], with specific activities related to tumor–stromal interactions and tumor suppression. NCAM1 forms a complex with N-cadherin and FGFRs to promote cell adhesion, affecting tumor progression in various ways [52]. Of the enriched downregulated genes, *SMO* is a critical actor in the regulation of the hedgehog pathway in PCa promotion [53], and the chemokine *CXCL1* promotes tumorigenic and angiogenic activity [54]. These genes, along with *PTX3*, could play an important role in the growth suppression of FGFRL1-KD tumors. The bioinformatics analysis of interactions between mouse-derived stromal and human PC3M tumor proteins using the CASTIN software [40] further indicated altered associations between the proteins regulating cell adhesion, bidirectional tumor stromal signaling, tumor growth, angiogenesis, and lymph angiogenesis in FGFRL1-KD versus control tumors, suggesting a fundamental rewiring of functional pathways and tumor–stromal contacts in xenografts.

## 5. Conclusions

Our results indicate that FGFRL1 can promote PCa growth and progression. The increased expression and cellular re-localization of FGFRL1 are associated with adverse clinicopathological characteristics. Importantly, nuclear FGFRL1 correlates with a shortened time to BCR, an observation that may have prognostic clinical value. Contrary to the increased growth of PCa in association with high FGFRL1 expression, our experimental results showed that FGFRL1 KD led to decreased xenograft growth. They also suggested that decreased membranous FGFRL1 in PCa cells can affect FGF signaling and disturb cellular differentiation. Our bioinformatics analyses strongly suggested that FGFRL1 functions primarily relate to tissue morphogenesis, differentiation, and modulation of the ECM and the regulation of tumor–stroma interactions that affect tumor growth [4,5,11], which are all hallmarks of FGF/FGFR functions [10,55].

## Figures and Tables

**Table 1:** Correlation of FGFRL1 staining with the Gleason score, pre-operative PSA, and Ki67 expression (Spearman rank correlation).

Variables	FGFRL1 Staining IHC Scores	Spearman Rank Correlation Coefficient	*p*-Value
	Membrane	−0.200	**0.018**
Gleason Score	Cytoplasm	0.217	**0.010**
	Nucleus	0.297	**0.000**
	Membrane	−0.004	0.966
Pre-operative PSA	Cytoplasm	−0.141	0.098
	Nucleus	0.199	**0.019**
	Membrane	−0.269	**0.002**
Ki67 IHC scores	Cytoplasm	0.261	**0.002**
	Nucleus	0.097	0.261

## Data Availability

Sequencing data files have been deposited in NCBI’s Gene Expression Omnibus (Edgar et al., 2002) and are accessible through GEO Series accession number GSE174148 for cell line and GSE174361 for xenograft data. Other data supporting the findings in this study are available upon reasonable request from the authors.

## Supplementary Materials

The following supporting information can be downloaded at: https://www.mdpi.com/2072-6694/14/2/278/s1, Figure S1: Uncropped Western blots referring to the ones included in the current article, Figure S2: FGFRL1 mRNA expression in prostate cancer, Figure S3: Immunohistochemical staining controls for FGFRL1, Figure S4: FGFRL1 expression in prostate cancer cell lines and characterization of transfected cells, Figure S5: The effects of FGFR inhibitors on PC3M FGFRL1-KD prostate cancer cells, Figure S6: FGFRL1 immunostaining of xenografts, Figure S7: Immunohistochemical and Western blot analyses of xenograft tumors, Figure S8: Validation of differential expressions of target genes FZD7 and PTX3 in xenografts formed by control versus FGFRL1-KD cells, Table S1: Association of FGFRL1 IHC staining and clinico-pathological features of primary PCa in TMA I (*n* = 139), Table S2: Univariable and multivariable Cox regression analysis of FGFRL1 staining and clinico-pathological parameters regarding to BCR-free survival in primary PCa, (*n* = 117, no neoadjuvant or adjuvant treatment), Table S3A: Genes significantly and reproducibly up-regulated in FGFRL1-KD cells—xenografts and 2D monolayer cultures (corresponding to Venn diagram in Figure 5C), Table S3B: Genes significantly and reproducibly downregulated in FGFRL1-KD cells—xenografts and 2D monolayer cultures, corresponding to Venn diagram shown in Figure 5C, Table S4A: Top 100 most prominently upregulated genes in mouse xenograft tumors after stable knockdown of FGFRL1, Table S4B: Top 100 most prominently downregulated genes in mouse xenograft tumors after stable knockdown of FGFRL1, Table S5: Significantly enriched tumor–stroma interactions detected by the CASTIN algorithm in PC3M xenografts derived from FGFRL1-KD versus control cells, Table S6: The sequencing coverage and quality statistics of next-generation sequencing (NGS) data for PC3M control and FGFRL1 knockdown cell line and xenografts, Supplementary Material and Methods.

## Abbreviations

AdjPr: adjacent benign prostate; ADT: androgen deprivation therapy; AdvPCa: advanced prostate cancer; AR: androgen receptor; BCR: biochemical recurrence; BPH: benign prostatic hyperplasia; COL6A2: collagen6A2; CRPC: castration-resistant prostate cancer; CTSB: cathepsin B; ECM: extracellular matrix; EGFP: enhanced green fluorescent protein; ESCC: esophageal squamous cell carcinoma; EthD1: Ethidium homodimer-1; FGF: fibroblast growth factor; FGF8: fibroblast growth factor 8; FGFRL1: fibroblast growth factor receptor-like 1; FRS2α: FGFR-substrate 2α; GO: Gene Ontology; GS: Gleason score; GSEA: gene set enrichment analysis; H&E: hematoxylin–eosin; HPIN: high-grade prostatic intraepithelial neoplasia; IHC: immunohistochemistry; KD: knockdown; NCBI: National Center for Biotechnology Information; PCa: prostate cancer; PH: proportional hazard; PTX3: pentraxin-3; MMP1/24: matrix metalloproteinase 1/24; PSM: positive surgical margins; PSA: prostate-specific antigen; qRT-PCR: quantitative real-time polymerase chain reaction; pTNM: pathological TNM stage; RP: radical prostatectomy; SEMA3B: semaphorin 3B; TBP: TATA-box binding protein; TMA: tissue microarray; χ^2^ test: Pearson’s chi-squared test.
