# Supplementary material for: Increased Expression and Altered Cellular Localization of Fibroblast Growth Factor Receptor-Like 1 (FGFRL1) Are Associated with Prostate Cancer Progression
Source: Cancers (Basel). 2022 Jan 7;14(2):278. doi: 10.3390/cancers14020278 (PMC8796033; doi:10.3390/cancers14020278)
Supplement: Supplementary file 1 [file cancers-14-00278-s001.zip › cancers-1502284-supplementary/Suppl Materials & Methods.pdf]

Supplementary Materials and Methods

Increased expression and altered cellular localization of fibroblast growth factor receptor like 1 (FGFRL1) are associated with prostate cancer progression

Lan Yu, Mervi Toriseva[†], Syeda Afshan[†], Mario Cangiano, Vidal Fey, Andrew Erickson, Heikki Seikkula, Kalle Alanen, Pekka Taimen, Otto Ettala, Martti Nurmi, Peter J. Boström, Markku Kallajoki, Johanna Tuomela, Tuomas Mirtti, Inès Beumer, Matthias Nees, Pirkko Härkönen.
[†] These authors contributed equally to this work.

1. TMA immunohistochemical staining and scoring

TMA blocks were cut into 3 µm thick sections, and a Lab Vision autostainer (Thermo Fisher) was used for hematoxylin-eosin (H&E) and immunohistochemical stainings as described previously[1]. Briefly, sections were deparaffinized and rehydrated in a graded series of treatments with xylene (3x5 min) 100% ethanol (2x2 min), 96% ethanol (2x2 min) and washed briefly ddH_2O. Antigen retrieval was in target retrieval buffer pH9, DAKO S2367), by heating in microwave (2x7 min). After cooling to room temperature, and washing in TBS buffer, also used in subsequent washings, the sections were treated with 3% hydrogen peroxide in methanol for 10 min at room temperature to block the endogenous peroxidase. After washing, nonspecific epitopes were blocked with antibody diluent (10 min, DAKO), followed by incubation with primary antibody, anti-FGFRL1 (Abcam Cat# ab95940, RRID:AB_10687283, 2µg/ml, 1:300) or anti-Ki67 (Agilent Cat# M7240, RRID:AB_2142367,1:200). The sections were then blocked for 20 min with post antibody blocking buffer (ImmunoLogic), and a two component detection system of peroxidase, goat antimouse/rabbit HRP (ImmunoLogic)was used to detect the antigen bound rabbit primary antibody complex (30 min incubation). The sections were washed. and incubated with peroxidase compatible chromogen according to manufacturer's protocol (K3468, DAKO).The sections were then washed with ddH_2O, counterstained with hematoxylin, washed again, dehydrated, and mounted on slides.

Human colon and liver sections (https://www.proteinatlas.org/ENSG00000127418-FGFRL1/tissue) included in each TMA slide were used as positive controls for immunohistochemical stainings with anti-FGFRL1 antibody. For negative controls of the anti-FGFRL1 immunostainings, anti-FGFRL1 antibody was replaced with normal (non-immune) rabbit serum or only with the buffer used to dilute primary antibody.

For analysis and scoring, digital images of H&E-, Ki67-, and FGFRL1-stained TMA sections were acquired using a Pannoramic scanner (3DHISTECH, Hungary). Representative areas of BPH, AdjPr, HGPIN, primary PCa, AdvPCa and CRPC were annotated by experienced pathologists, and FGFRL1 staining was evaluated by IHC score. Cell membrane, cytoplasmic and nuclear FGFRL1 staining were evaluated separately. The IHC score of cytoplasmic staining represents the sum of the IHC intensity levels (Int) observed, multiplied by the number of corresponding positive percentages (%Pos) (IHC score = Int1*%Pos+ Int2*%Pos+Int3*%Pos). FGFRL1 IHC scores were calculated exclusively from luminal epithelium or cancer cells. The membrane and nuclear IHC stainings were scored as positive or negative. The mean IHC score of parallel cores (up to four cores per sample) was used to represent a single patient. Staining of stromal and atrophic areas was not analyzed in this study. The IHC images were evaluated by three persons (Y.L., A.E or P.H.) independently, blinded from the clinical patient status. Cases with disagreement were re-evaluated with pathologist until consensus was reached.

2. Western blotting

Cells were lysed in RIPA buffer containing Protease and Phosphatase inhibitors (Pierce Ltd.) for protein examination. Xenograft samples were homogenized in RIPA buffer, also containing protease and phosphatase inhibitors. Protein concentration was measured using the standard Bicinchoninic Acid (BCA) assay (BioRad). Samples were heated to 95°C for 5 min, separated on a 10% SDS-polyacrylamide gel and transferred to Nitrocellulose Pure Transfer Membrane (Santa Cruz Biotechnology Ltd). The membranes were blocked with 5% non-fat milk in TBS-Tween buffer for 1 hour at room temperature, and incubated overnight with primary antibody at 4°C. After triple TBS-Tween washing, the membranes were incubated with the secondary antibody (LI-COR Biosciences Cat# 925-32213, RRID:AB_2715510 or LI-COR Biosciences Cat# 925-68072, RRID:AB_2814912, 1:2500) for 1 hour at room temperature. Signals were detected with the Odyssey Infrared Imaging System (Li-COR), except for P-FRS2 and FRS2, which were detected using HRP-conjugated secondary antibodies and enhanced chemiluminescence on film. Anti-alpha tubulin (Abcam Cat# ab4074, RRID:AB_2288001) was used as a loading control. Antibodies used in this study include the rabbit polyclonal anti-FGFRL1 raised against recombinant protein fragment of the amino acids 150-168 of human FGFRL1 (Abcam Cat# ab95940, RRID:AB_10687283), anti-phospho-ERK1/2 (Cell Signalling

Technology Cat# 9101, RRID: AB_331646), anti-ERK1/2 (Cell Signaling Technology Cat# 9102, RRID:AB_330744), anti-phospho-FRS2-α (Cell Signaling Technology Cat# 3864, RRID:AB_2106222), anti-FRS2 (Santa Cruz Biotechnology Cat# sc-8318, RRID:AB_2106228), anti-phospho-AKT Ser473 (Santa Cruz Biotechnology Cat# sc-7985, RRID:AB_667741), anti-AKT (Cell Signaling Technology Cat# 9275, RRID:AB_329828), anti-CDK2 (Santa Cruz Biotechnology Cat# sc-163, RRID:AB_631215), anti-CDK4 (Santa Cruz Biotechnology Cat# sc-23896, RRID:AB_627239), anti-CDK6 (Santa Cruz Biotechnology Cat# sc-7181, RRID:AB_2076995), anti-cyclin E (Santa Cruz Biotechnology Cat# sc-481, RRID:AB_2275345), and anti-cyclin D1 (Abcam Cat# ab16663, RRID:AB_443423). Densitometry analysis of Western blot bands was performed with the Image studio software (LI-COR BioSciences) for Figure S4 (xenograft protein lysates) and with ImageJ for other Western blots.

3. Immunofluorescence (IF) staining of FGFRL1

Cells were plated on cover slips in the growth medium. The next day, they were fixed and simultaneously permeabilized with PBS containing 2% paraformaldehyde and 0.5% Triton X-100 for 10 minutes. After blocking in 10% goat serum, the cells were incubated with anti-FGFRL1 primary antibody (Abcam Cat# ab95940, RRID:AB_10687283, 1:100) over night in +4°C, washed and labeled with anti-rabbit secondary antibody for 1 hr (Thermo Fisher Scientific Cat# A32731, RRID:AB_2633280 ,1:200). Samples were mounted with ProLong Gold Antifade Reagent (Invitrogen). The knockdown cells were imaged using Perkin Elmer Operetta, confocal mode and 63x objective. The FGFRL1 overexpressing cells were imaged with an Axiovert-200M microscope equipped with a Yokogawa CSU22 spinning disc confocal unit and a Plan-Neofluar 40x objective (Carl Zeiss Microimaging GmbH, Oberkochen, Germany). Fluorescent probe Draq5 (Thermo Fisher Scientific REF# 62251) was used to visualize the nuclei.

4. Immunohistochemical (IHC) staining of xenografts

Formalin-fixed and paraffin-embedded xenograft blocks were cut into 5 µm sections for H&E and immunohistochemical stainings. These sections were deparaffinized in xylene and rehydrated with graded ethanol. The slides were cooked in 10 mM sodium citrate buffer (pH 6) in a microwave oven for 10 min for antigen retrieval, followed by immersion in 3% H_2O_2 in

methanol for blocking of endogenous peroxidase activities. After washing with PBS (pH 7.4), normal horse (2%) or rabbit serum (10%) were applied for 30 min at room temperature (RT) to block non-specific binding. Samples were then incubated overnight at 4°C with primary antibodies using optimized dilutions. After washing, secondary antibodies biotinylated goat anti-rabbit or rabbit anti-rat (Vector Laboratories Cat# BA-1000, RRID:AB_2313606 or Vector Laboratories Cat# BA-4000, RRID:AB_2336206, 1:200) were applied for 1 hour at RT. Slides were incubated with avidin-biotin complex (Vector Laboratories Cat# PK-4000, RRID:AB_2336818) for 1 hour at RT. Detection was performed with the DAB kit (Vector Laboratories Cat# SK-4100, RRID:AB_2336382), according to the manufacturer's instructions. Samples were counterstained with Mayer's hematoxylin, dehydrated and mounted. An anti-phospho-histone H3 (PHH3) antibody (Cell Signaling Technology Cat#9701, RRID:AB_331535,1:200) was used to study the proportion of mitotic cells and a CD34 antibody (Santa Cruz Biotechnology Cat# sc-18917, RRID:AB_2260027, 1:50) was used to determine the blood vessel density in xenograft tumors. All sections were scanned with a Pannoramic 250 slide scanner (3DHISTECH, Hungary). For PHH3 analysis, four images were collected at 20x magnification from one xenograft. Positive PHH3 staining cells and total cell number were counted and the percentage of PHH3 positive cells was calculated. The combined length of all CD34 stained blood vessels and total tumor area were measured with the Image J software (RRID:SCR 003070) and used to evaluate the blood vessel density.

5. Next generation sequencing (NGS)

Total RNA was harvested from PC-3M ctrl-KD and PC-3M FGFRL1-KD cell clones and their respective subcutaneous xenografts in athymic nu/nu mice. RNA quality control, library preparation, and next generation sequencing (NGS) were performed for cell line RNAs by The Finnish Microarray and Sequencing Centre (FMSC) at Turku Biosciences, University of Turku and Åbo Akademi, Finland, and for xenograft RNA by GenomeScan, Leiden, The Netherlands. In both approaches, RNA quality was detected by an Agilent Bioanalyzer 2100. Library preparation for RNA sequencing (RNAseq) of cell lines was done according to the "Illumina TruSeq Stranded mRNA Sample Preparation Guide", based on the enrichment of poly-A containing mRNA. The excellent and equal quality of the libraries was confirmed with Advanced Analytical Fragment Analyzer and the concentrations of the libraries were

quantified with Qubit® Fluorometric Quantitation (Life Technologies). All samples were subjected to single-end sequencing with 50bp read length, using an Illumina HiSeq 2500 with TruSeq v3 sequencing chemistry (Kinghorn Centre for Clinical Genomics (KCCG), Sydney, NSW, AU). Technical quality of the run was good and the cluster amount was as expected. Greater than 96 % of all bases above Q30 was achieved. The base calling was performed using Illumina's standard bcl2fastq software. Total RNA extracted from mouse xenografts was sequenced by GenomeScan. Samples were processed using the NEB Next Ultra II Directional RNA Library Prep Kit for Illumina, following protocol NEB #E7760S/L. Briefly, mRNA was isolated from total RNA using oligo-dT magnetic beads. After fragmentation of mRNA, cDNA synthesis was performed. The product was used for ligation with sequencing adapters, followed by PCR amplification. Quality and yield of cDNA after sample preparation were measured with Agilent Bioanalyzer 2100 Fragment Analyzer, validating that the resulting products were consistent with the expected size distribution (300-500 bp). DNA sequencing was performed on a NovaSeq6000 (150bp paired end method) with *NovaSeq control software NCS v1.6*, according to manufacturer's protocols, using a concentration of 1.1 nM of cDNA. Image analysis, base calling, and quality check were performed with the *Illumina data analysis pipeline RTA3.4.4* and the program *Bcl2fastq v2.20* (RRID:SCR_015058) as shown in supplementary table 6.

6. RNAseq data analysis

For RNAseq analysis of the cell lines, the workflow was written in *R v3.3* (https://www.R-project.org/), where Unix command line interface (CLI) tools were called by means of dedicated wrapper functions. Sequence quality was assessed with *FastQC v0.11.2* (RRID:SCR_014583) and reads were aligned to the reference genome version GRCh38.81 using *TopHat2 v2.1.0*. Alignment files were sorted and converted for down-stream analysis using *SAM v1.3* tools (RRID:SCR_010951) and reads were counted using *HTSeq v0.6.1p1* (RRID:SCR_005514). Subsequent data analysis was performed using standard *R/Bioconductor*, and statistical assessment of differential gene expression was done using the limma package v3.28.21 (RRID:SCR_010943), following the principles and pipeline published by Anders et al. 2013.[2, 3] The log2 fold-changes and adjusted p-values of the differentially expressed genes between the PC-3M control-KD (n=4) and FGFRL1-KD clones (n=2) were extracted after

applying voom[4] with sample quality weights and quantile-normalization to the filtered counts. [3]

For the analysis of the 10 mouse xenograft samples, which contained both human and mouse DNA reads, paired end fastq files were first trimmed of adapter sequences using the 'ILLUMINACLIP' step from '*Trimmomatic' version 0.36*. Alignment of reads to GRCh37.75 human reference and GRCm38.p4 mouse reference genome built, respectively, were performed with *TopHat v2.0.14* (RRID:SCR_013035). Next, disambiguation of human/mouse reads was performed with *Disambiguate 1.0*. Calculation of gene level raw counts was performed using '*HTSeq' version 0.9.1* and GRCh37.75/ GRCm38.p4 annotation files (.gtf) for human and mouse reads respectively and independently. Eventually, gene and transcripts level FPKM quantification was done using *cufflinks-2.2.1* and GRCh37.75/GRCm38.p4 gtf files for human and mouse reads respectively. The R package '*Deseq2' v1.22.2* was used with default parameters to calculate differentially expressed genes between FGFRL1-KD vs. control xenografts, starting from raw counts. Differentially expressed genes (from both human and mouse reads) were identified using the threshold of adjusted p-value of 0.05. Statistical analyses and data visualization were performed within '*R*' *v3.5.0* and the R packages 'heatmap3' v1.1.6 and '*GViz*' *v1.30.1*. To assess the biological relevance of differentially expressed genes (DE, log fold change > 2 fold with FDR of < 0.001), we explored functional pathways and gene sets enriched in these gene lists. DE gene lists were analyzed using the Gene Set Enrichment Analysis (GSEA) tool (www.gsea-msigdb.org), provided by BROAD Institute/MIT, and visualized using Metascape (RRID:SCR_016620).

7. Ligand-receptor analyses for xenograft tumors (CASTIN)

The Castin (Cancer-Stromal Interactome analysis) pipeline[5] has been used with default parameters to unveil ligand-receptors interactions between mouse stroma and human tumor cells. Specifically, raw fastq files of each sample were mapped to a human/mouse mixed reference genome (hg38/mm10) to generate normalized human/mouse gene counts and ligand-receptor pairs interaction strength. A t-test has been applied to the observed interaction strengths between the two groups (FGFRL1-KD vs. control-KD) and a significance level of 0.05 was chosen.

References

1. Yu L, Toriseva M, Tuomala M, Seikkula H, Elo T, Tuomela J, Kallajoki M, Mirtti T, Taimen P, Boström PJ. Increased expression of fibroblast growth factor 13 in prostate cancer is associated with shortened time to biochemical recurrence after radical prostatectomy. *Int J Cancer.* 2016; **139**: 140-52.

2. Ritchie ME, Phipson B, Wu D, Hu Y, Law CW, Shi W, Smyth GK. limma powers differential expression analyses for RNA-sequencing and microarray studies. *Nucleic Acids Res* 2015; **43**: e47.

3. Anders S, McCarthy DJ, Chen Y, Okoniewski M, Smyth GK, Huber W, Robinson MD. Count-based differential expression analysis of RNA sequencing data using R and Bioconductor. *Nat Protoc* 2013; **8**: 1765-86.

4. Law CW, Chen Y, Shi W, Smyth GK. voom: Precision weights unlock linear model analysis tools for RNA-seq read counts. *Genome Biol* 2014; **15**: R29-r29.

5. Komura D, Isagawa T, Kishi K, Suzuki R, Sato R, Tanaka M, Katoh H, Yamamoto S, Tatsuno K, Fukayama M, Aburatani H, Ishikawa S. CASTIN: a system for comprehensive analysis of cancer-stromal interactome. *BMC Genomics* 2016 ;**17**: 899-z.
