# Supplementary material for: Increased Expression and Altered Cellular Localization of Fibroblast Growth Factor Receptor-Like 1 (FGFRL1) Are Associated with Prostate Cancer Progression
Source: Cancers (Basel). 2022 Jan 7;14(2):278. doi: 10.3390/cancers14020278 (PMC8796033; doi:10.3390/cancers14020278)
Supplement: Supplementary file 1 [file cancers-14-00278-s001.zip › cancers-1502284-supplementary/Supplementary Figures updated Figure S1.pdf]

Supplementary Figures

Increased expression and altered cellular localization of fibroblast growth factor receptor like 1 (FGFRL1) are associated with prostate cancer progression

Lan Yu, Mervi Toriseva†, Syeda Afshan†, Mario Cangiano, Vidal Fey, Andrew Erickson, Heikki Seikkula, Kalle Alanen, Pekka Taimen, Otto Ettala, Martti Nurmi, Peter J. Boström, Markku Kallajoki, Johanna Tuomela, Tuomas Mirtti, Inès Beumer, Matthias Nees, Pirkko Härkönen.
† These authors contributed equally to this work.

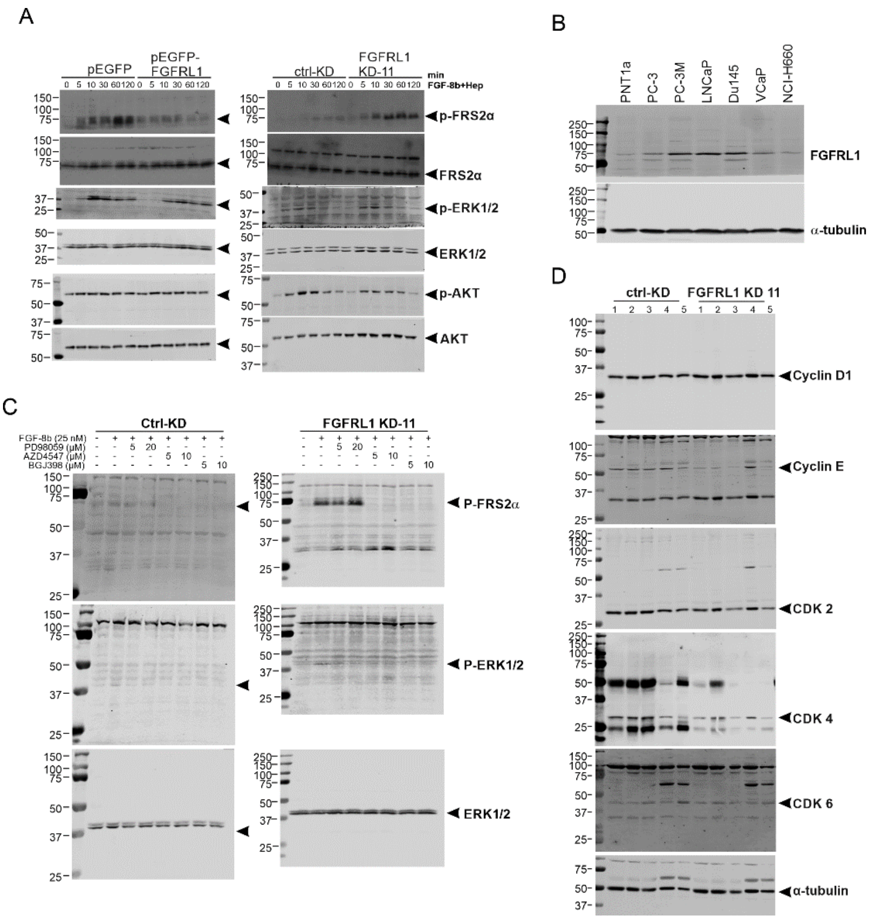

Figure S1. Uncropped western blots referring to the ones included in the current article. A) Figure 3, B) Figure S4, C) Figure S5, and D) Figure S7.

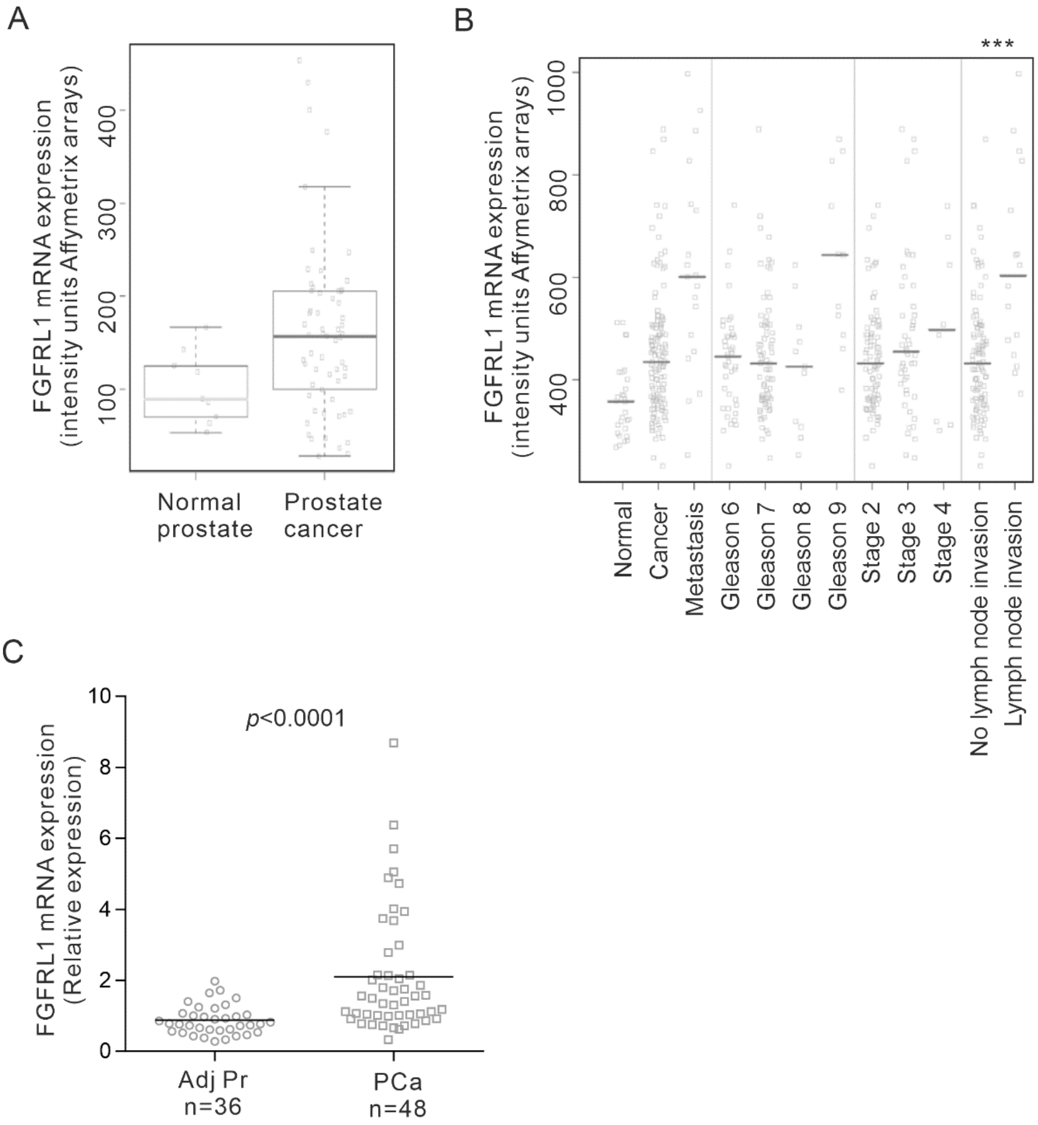

Figure S2. FGFRL1 mRNA expression in prostate cancer. (A), In silico analysis of FGFRL1 mRNA expression in normal prostate tissues and prostate adenocarcinomas in the IST dataset (Medisapiens Oy, Helsinki). (B), Detailed in silico analysis of FGFRL1 mRNA expression and correlation with different clinical parameters in the MSKCC 2010 data set. ***p < 0.001. (C), Relative mRNA expression of FGFRL1 was analyzed in adjacent benign prostate (AdjPr) and cancerous prostate (PCa) by qPCR from specimens containing >50% of corresponding epithelium.

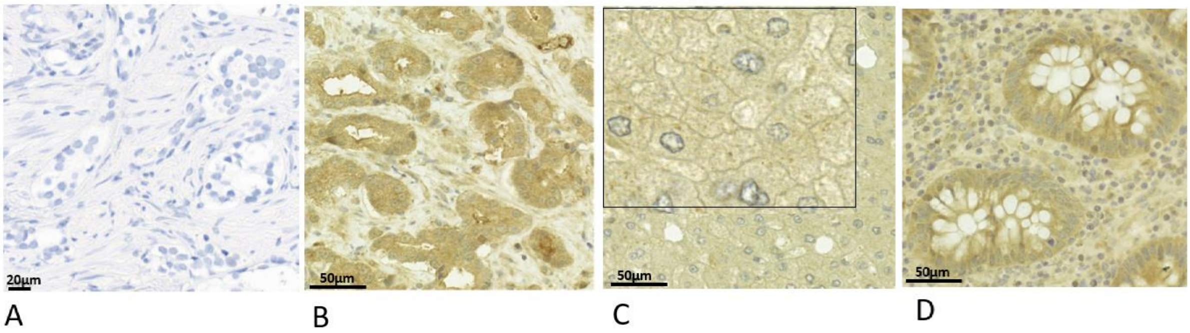

Figure S3. Immunohistochemical staining controls for FGFRL1. (A), Negative control (primary antibody ab95940 omitted). (B), Example of strong immunostaining, tissue sample from prostate cancer specimen. (C-D), Positive controls used in tissue microarrays (TMAs). Liver, cytoplasmic and membranous (inset) staining of hepatocytes (C) and staining of colon (D).

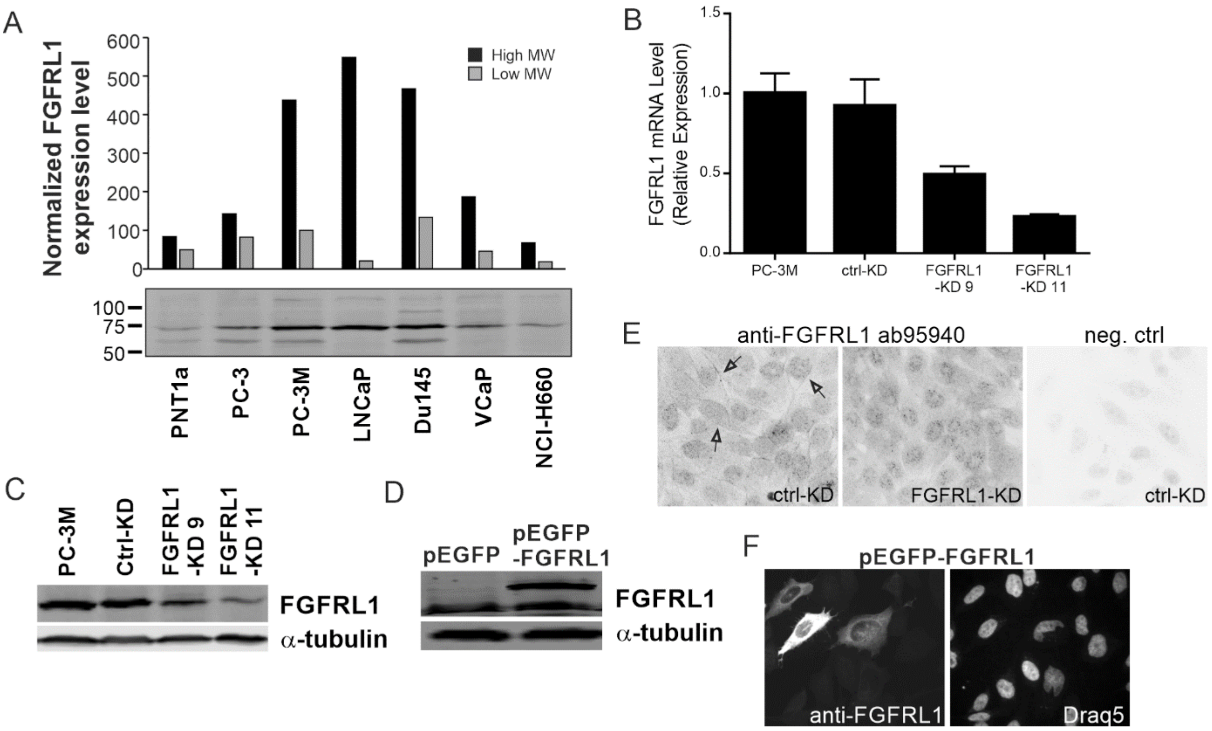

Figure S4. FGFRL1 expression in prostate cancer cell lines and characterization of transfected cells. (A), Prostate cancer cell line lysates were analyzed for FGFRL1 expression by Western blot. Alpha-tubulin was used as a loading control. (B-C), FGFRL1 mRNA (B) and protein (C) levels were analyzed in parental PC3M cells, knockdown mock-transfected cells (ctrl-KD), and FGFRL1 knockdown cells (FGFRL1-KD 9 and 11) using qRT-PCR and Western blot, respectively. (D), Protein levels of FGFRL1 were analyzed in pEGFP-control vector and pEGFP-FGFRL1-expression vector transfected PC3M cells by Western blot. Western blot signal intensities were quantitated with ImageJ and relative signal levels are shown below the blot. (E), Endogenous expression of FGFRL1 was detected in ctrl-KD and FGFRL1-KD cells with immunofluorescence staining (arrows indicate FGFRL1 staining at cell membranes in ctrl-KD cells). Objective 63x. (F), Immunofluorescence staining of FGFRL1 protein in pEGFP-FGFRL1-expression vector transfected PC3M cells. Draq5 was used to stain the nuclei. Objective 40x.

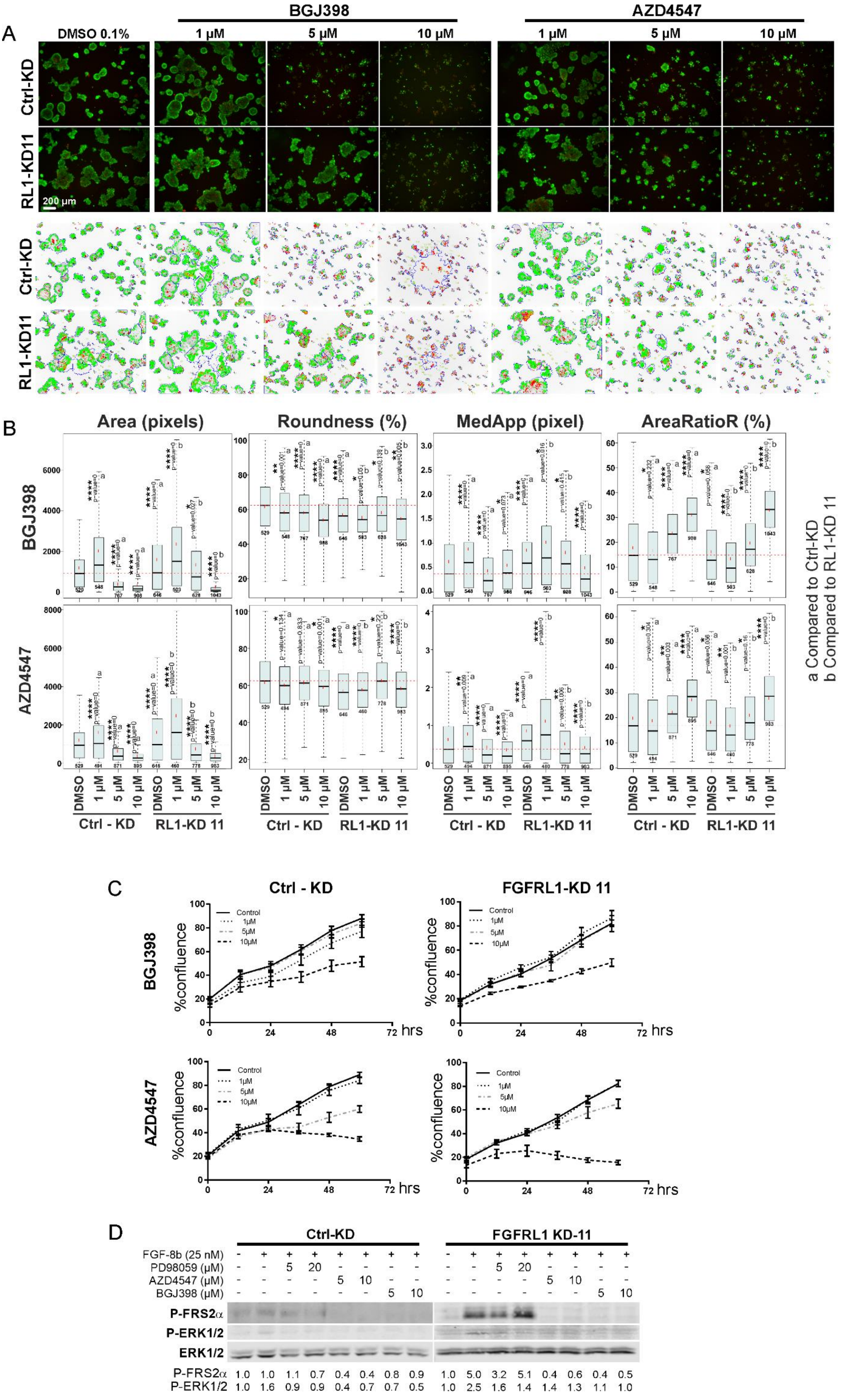

A
BGJ398
AZD4547
DMSO 0.1%
1 μM
5 μM
10 μM
Ctrl-KD
RL1-KD11
200 μm
B
Area (pixels)
Roundness (%)
MedApp (pixel)
AreaRatioR (%)
BGJ398
AZD4547
DMSO
1 μM
5 μM
10 μM
Ctrl - KD
RL1-KD 11
a Compared to Ctrl-KD
b Compared to RL1-KD 11
C
Ctrl - KD
FGFRL1-KD 11
BGJ398
AZD4547
%confluence
hrs
Control
1μM
5μM
10μM
D
Ctrl-KD
FGFRL1 KD-11
FGF-8b (25 nM)
PD98059 (μM)
AZD4547 (μM)
BGJ398 (μM)
P-FRS2α
P-ERK1/2
ERK1/2
P-FRS2α 1.0 1.0 1.1 0.7 0.4 0.4 0.8 0.9 1.0 5.0 3.2 5.1 0.4 0.6 0.4 0.5
P-ERK1/2 1.0 1.6 0.9 0.9 0.4 0.7 0.7 0.5 1.0 2.5 1.6 1.4 1.4 1.3 1.1 1.0

Figure S5. The effects of FGFR inhibitors on PC3M FGFRL1-KD prostate cancer cells. (A), Ctrl-KD and FGFRL1-KD cells were cultured in organotypic 3D cultures in the presence of 5% FBS and BGJ398, AZD4547 or 0.1% DMSO as a control (preculture 4 days and treated for 8 days). Cells were stained with Calcein-AM (green) and Ethidiumhomodimer-1 (red) for living and dead cells, respectively, and imaged using confocal microscope. Representative confocal image stack projections (upper rows) and image segmentation by AMIDA software (lower rows) are shown. (B), Quantitative morphometric image analysis of phenotypic features observed in 3D cultures was performed using AMIDA. Bonferroni-corrected *t*-tests (* $p<0.05$, ** $p<0.01$, *** $p<0.001$, **** $p<0.0001$). The total number of analyzed objects (in 6 replicate wells) is indicated under the whisker of the box and whisker plots. (C), Cell proliferation of ctrl-KD and FGFRL1-KD cells *in vitro* in 2D cultures, treated with BGJ398 and AZD4547 in the presence of 5% FBS. (D), Western blot analysis of FRS2α and ERK1/2 phosphorylation in ctrl-KD and FGFRL1-KD cells upon treatment with the MEK1/2 inhibitor PD98059 and FGFR inhibitors AZD4547 and BGJ398. Total ERK1/2 was used as a loading control. Note the enhanced signalling response to FGF-treatment in FGFRL1-KD cells. Western blot signal intensities were quantitated with ImageJ and relative signal levels are shown below the blot.

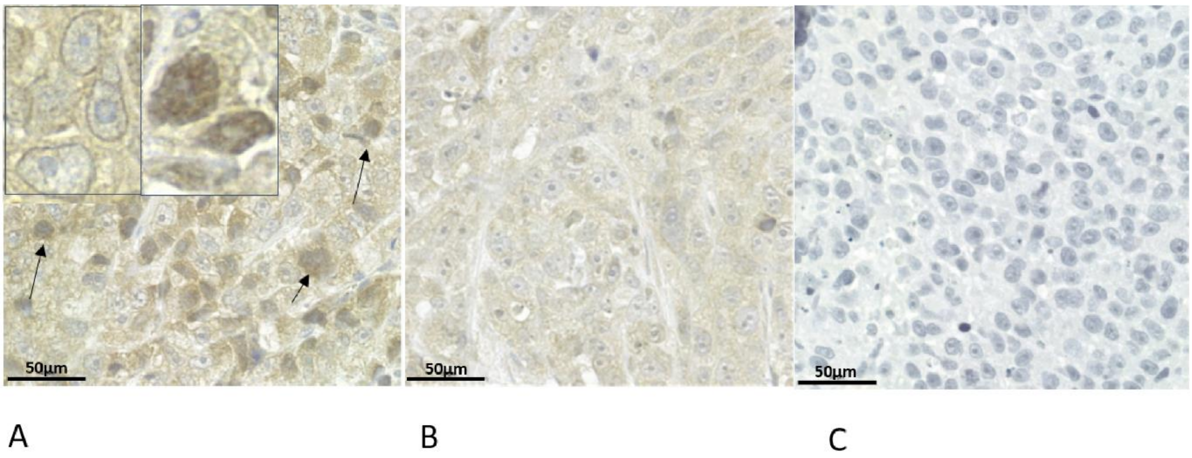

Figure S6. FGFRL1 immunostaining of xenografts. (A), FGFRL1 (control) xenografts (insets show examples of membranous staining and strong cytoplasmic staining, arrows nuclear staining). (B), FGFRL1-KD xenografts. (C), Negative control (primary antibody ab 95940 omitted)

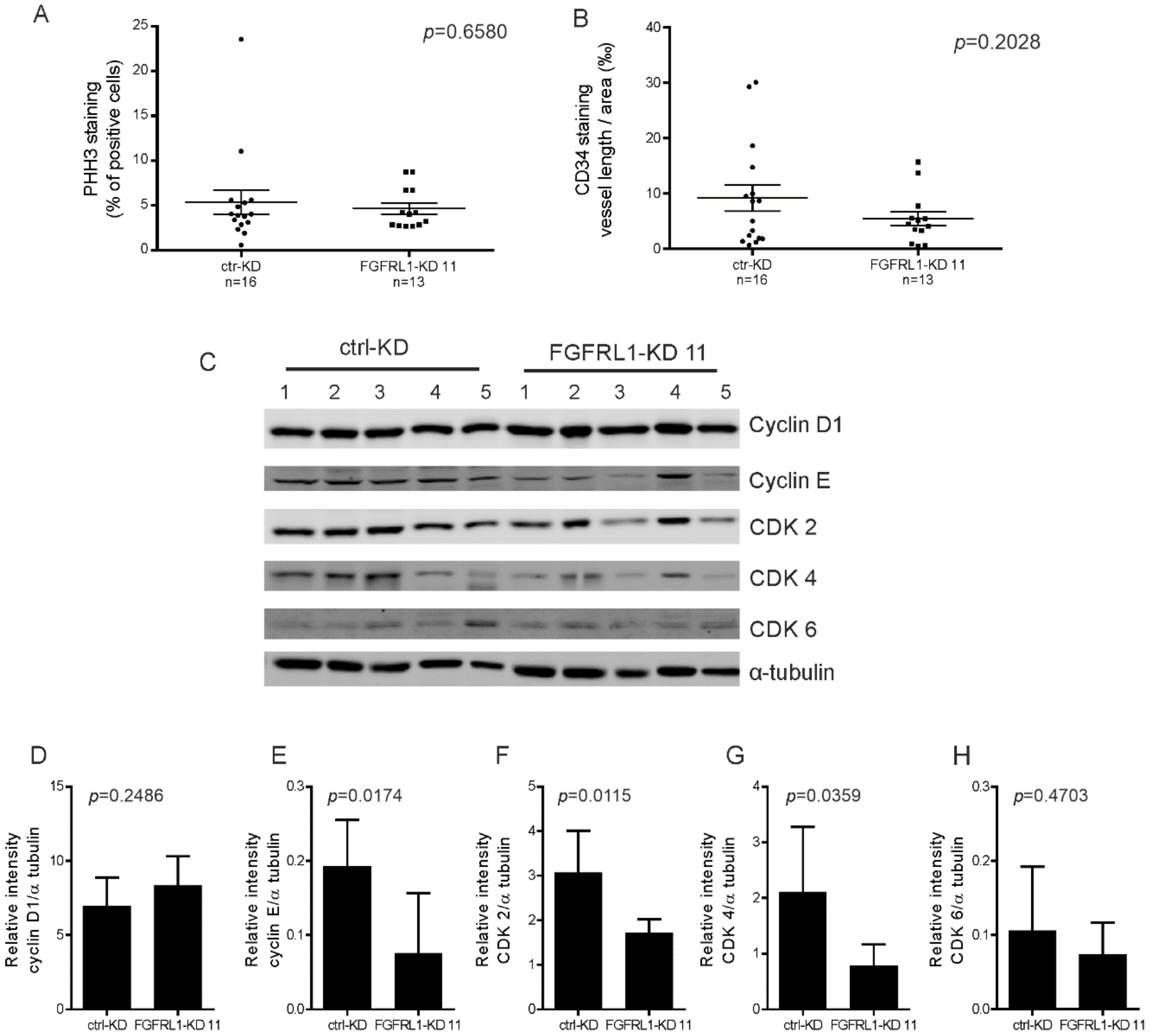

Figure S7. Immunohistochemical and Western blot analyses of xenograft tumors. (A), Percentage of positive PHH3-stained cells and (B), CD34-stained capillaries in xenografts. Four images of each xenograft were analyzed to determine the proportion of PHH3 positive tumor cells. Length of all CD34-stained blood vessels was compared to total tumor area. (C), Tumor lysates from 5 ctrl-KD and 5 FGFRL1-KD xenografts were used to examine cell cycle-related proteins. Alpha-tubulin was used as a loading control. (D-H), Intensities of cyclin D1, cyclin E, CDK2, 4, 6 bands were measured and related to the loading control α-tubulin. t-test was used in statistical analysis

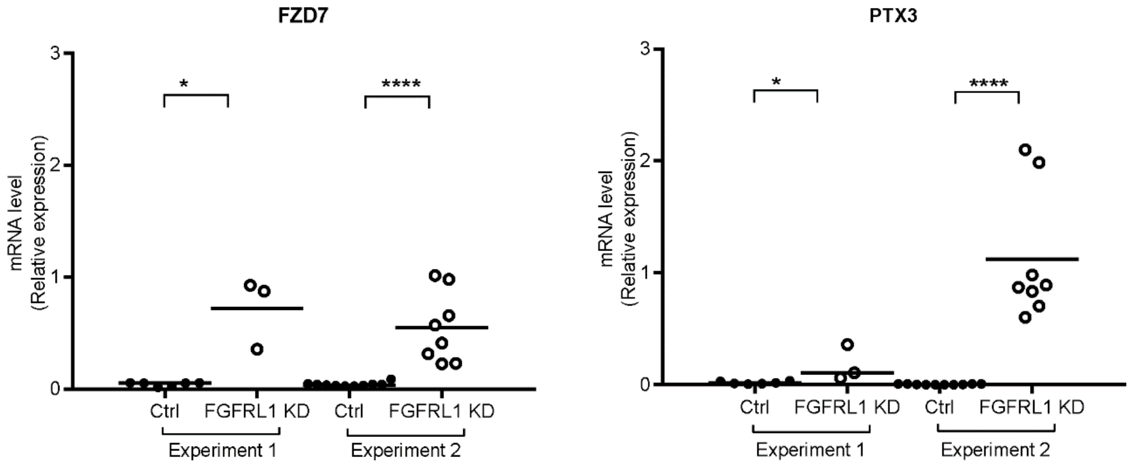

Figure S8. Validation of differential expressions of target genes FZD7 and PTX3 in xenografts formed by control versus FGFRL1-KD cells. Expression was determined in 2 independent experiments with 9 (6 control, 3 KD) and 18 mice (10 control, 8 KD), respectively. *p<0.05, ****p<0.0001.
