# Supplementary material for: Increased Expression and Altered Cellular Localization of Fibroblast Growth Factor Receptor-Like 1 (FGFRL1) Are Associated with Prostate Cancer Progression
Source: Cancers (Basel). 2022 Jan 7;14(2):278. doi: 10.3390/cancers14020278 (PMC8796033; doi:10.3390/cancers14020278)
Supplement: Supplementary file 1 [file cancers-14-00278-s001.zip › cancers-1502284-supplementary/Supplementary tables.pdf]

Supplementary Tables

Increased expression and altered cellular localization of fibroblast growth factor receptor like 1 (FGFRL1) are associated with prostate cancer progression

Lan Yu, Mervi Toriseva[†], Syeda Afshan[†], Mario Cangiano, Vidal Fey, Andrew Erickson, Heikki Seikkula, Kalle Alanen, Pekka Taimen, Otto Ettala, Martti Nurmi, Peter J. Boström, Markku Kallajoki, Johanna Tuomela, Tuomas Mirtti, Inès Beumer, Matthias Nees, Pirkko Härkönen.
[†] These authors contributed equally to this work.

Table S1: Association of FGFRL1 IHC staining and clinico-pathological features of primary PCa in TMA I (n=139).

		Membranous Staining			Cytoplasmic Staining			Nuclear Staining		
	No. of patients (%)	FGFRL1 low	FGFRL1 high	*p* value	FGFRL1 low	FGFRL1 high	*p* value	FGFRL1 low	FGFRL1 high	*p* value
Age (years)										
< 65	88(63)	45(51)	43(49)	0.668	45(51)	43(49)	0.643	58(66)	30(34)	0.570
≥ 65	51(37)	28(55)	23(45)		24(47)	27(53)		36(71)	15(29)	
Gleason score										
≤7	117(84)	56(48)	61(52)	**0.011**	60(51)	57(49)	0.372	86(74)	31(26)	**0.001**
8-9	22(16)	17(77)	5(23)		9(41)	13(59)		8(36)	22(64)	
pre-operative PSA (ng/ml)										
<10	87(63)	43(49)	44(51)	0.345	43(49)	44(51)	0.948	65(75)	22(25)	**0.021**
≥10	52(37)	30(58)	22(42)		26(50)	26(50)		29(56)	23(44)	
pT-category										
pT2a-T2c	59(42)	30(51)	29(49)	0.735	28(47)	31(53)	0.658	44(75)	15(25)	0.133
pT3a-T4	80(58)	43(54)	37(46)		41(51)	39(49)		50(63)	30(37)	
Positive surgical margin (PSM)										
NO	74(53)	39(53)	35(47)	0.963	38(51)	36(49)	0.667	56(76)	18(24)	**0.030**
YES	65(47)	34(52)	31(48)		31(48)	34(52)		38(58)	27(42)	

Table S2: Univariable and multivariable Cox regression analysis of FGFRL1 staining and clinico-pathological parameters regarding to BCR-free survival in primary PCa,
(n = 117, no neoadjuvant or adjuvant treatment)

	**Univariate**		**Multivariate**	
	**HR (95%)**	***P***	**HR (95%)**	***P***
FGFRL1 plasma membrane (high *vs.* low)	1.721 (0.848-3.490)	0.133		
FGFRL1 cytoplasm (low *vs.* high)	1.797 (0.886-3.644)	0.104		
FGFRL1 nucleus (low *vs.* high)	3.486 (1.732-7.017)	**<0.0001**	3.535 (1.747-7.153)	**<0.0001**
Age (<65 *vs.* ≥65)	1.346 (0.668-2.709)	0.406		
Preoperative PSA (<10ng/mL *vs.* ≥10ng/mL)	1.140 (0.557-2.333)	0.720		
Gleason score (≤7 *vs.* >8)	2.137 (0.923-4.946)	0.076	2.186 (0.937-5.100)	0.070
pT-category (T2 *vs.* T3)	1.388 (0.678-2.842)	0.370		
Surgical margin (negative *vs.* positive)	0.867 (0.428-1.757)	0.693		

Table S3A: Genes significantly and reproducibly up-regulated in FGFRL1-KD cells – xenografts and 2D monolayer cultures (corresponding to Venn diagram in Fig. 5C).

ENSEMBL GENE_ID	SYMBOL	GENE NAME	logFC xenografts	p-value	p-value adjusted	logFC cell line	p-value	p-value adjusted
ENSG00000087495	**PHACTR3**	phosphatase and actin regulator 3	**8.01**	5.14E-37	2.58E-34	**8.73**	7.30E-06	3.15E-03
ENSG00000160183	**TMPRSS3**	transmembrane protease, serine 3	**11.53**	3.39E-37	1.81E-34	**8.25**	9.06E-10	1.16E-05
ENSG00000155760	**FZD7**	frizzled class receptor 7	**6.35**	1.76E-22	2.30E-20	**5.77**	1.83E-08	5.09E-05
ENSG00000160185	**UBASH3A**	ubiquitin associated and SH3 domain containing A	**5.95**	5.02E-07	5.75E-06	**5.57**	6.59E-08	1.02E-04
ENSG00000131398	**KCNC3**	potassium voltage-gated channel subfamily C member 3	**3.77**	4.81E-51	6.13E-48	**5.49**	1.07E-05	3.79E-03
ENSG00000198223	**CSF2RA**	colony stimulating factor 2 receptor alpha subunit	**6.58**	6.65E-13	2.33E-11	**5.47**	8.12E-09	5.09E-05
ENSG00000160182	**TFF1**	trefoil factor 1	**1.71**	1.09E-03	5.31E-03	**5.46**	1.89E-07	2.19E-04
ENSG00000071073	**MGAT4A**	mannosyl -glycoprotein beta-1,4-N-acetylglucosaminyl transferase, isozyme A	**7.91**	2.28E-31	6.59E-29	**5.41**	3.30E-04	2.58E-02
ENSG00000176788	**BASP1**	brain abundant membrane attached signal protein 1	**4.31**	1.13E-15	5.98E-14	**5.32**	4.51E-06	2.22E-03
ENSG00000077274	**CAPN6**	calpain 6	**3.08**	5.35E-04	2.88E-03	**5.29**	1.03E-07	1.32E-04
ENSG00000177675	**CD163L1**	CD163 molecule like 1	**4.04**	7.07E-31	1.97E-28	**5.27**	2.52E-06	1.70E-03
ENSG00000186310	**NAP1L3**	nucleosome assembly protein 1 like 3	**5.57**	7.62E-40	4.59E-37	**4.64**	1.23E-04	1.59E-02
ENSG00000126752	**SSX1**	SSX family member 1	**4.22**	5.04E-11	1.31E-09	**4.62**	8.91E-06	3.56E-03
ENSG00000148082	**SHC3**	SHC adaptor protein 3	**5.14**	4.16E-68	1.29E-64	**4.54**	1.75E-06	1.40E-03
ENSG00000179083	**FAM133A**	family with sequence similarity 133 member A	**4.11**	9.05E-33	3.07E-30	**4.48**	1.29E-04	1.64E-02
ENSG00000163347	**CLDN1**	claudin 1	**3.33**	8.01E-26	1.35E-23	**4.3**	1.21E-08	5.09E-05
ENSG00000185973	**TMLHE**	trimethyllysine hydroxylase, epsilon	**2.63**	3.86E-29	9.30E-27	**4.25**	5.01E-05	9.49E-03
ENSG00000237442	**HNRNPA1P57**	HNRNPA1P57	**1.74**	7.91E-04	4.05E-03	**4.15**	3.19E-05	7.03E-03
ENSG00000183287	**CCBE1**	collagen and calcium binding EGF domains 1	**4.99**	3.93E-14	1.66E-12	**4.03**	1.66E-04	1.86E-02
ENSG00000078725	**BRINP1**	BMP/retinoic acid inducible neural specific 1	**5.64**	4.39E-12	1.35E-10	**3.57**	5.99E-06	2.83E-03
ENSG00000196220	**SRGAP3**	SLIT-ROBO Rho GTPase activating protein 3	**3.14**	6.88E-24	1.03E-21	**3.53**	1.32E-06	1.12E-03
ENSG00000169083	**AR**	androgen receptor	**7.77**	2.45E-11	6.67E-10	**3.45**	3.34E-05	7.24E-03
ENSG00000155962	**CLIC2**	chloride intracellular channel 2	**2.99**	4.84E-14	2.02E-12	**3.21**	4.97E-05	9.49E-03
ENSG00000125966	**MMP24**	matrix metallopeptidase 24	**1.7**	1.54E-04	9.74E-04	**3.16**	7.94E-05	1.20E-02
ENSG00000196611	**MMP1**	matrix metallopeptidase 1	**2.58**	1.43E-14	6.50E-13	**3.05**	6.51E-04	3.81E-02
ENSG00000163661	**PTX3**	pentraxin 3	**5.55**	4.21E-24	6.47E-22	**2.84**	1.16E-03	4.97E-02
ENSG00000134193	**REG4**	regenerating family member 4	**2.22**	3.65E-04	2.07E-03	**2.47**	8.15E-05	1.20E-02
ENSG00000198865	**CCDC152**	coiled-coil domain containing 152	**3.51**	4.35E-10	9.50E-09	**2.41**	2.42E-05	6.32E-03
ENSG00000140297	**GCNT3**	glucosaminyl transferase 3, mucin type	**2.2**	2.45E-10	5.54E-09	**2.31**	4.49E-04	3.05E-02
ENSG00000102271	**KLHL4**	kelch like family member 4	**2.71**	1.41E-21	1.57E-19	**2.21**	9.20E-06	3.56E-03
ENSG00000043355	**ZIC2**	Zic family member 2	**3.1**	2.20E-13	8.33E-12	**2.18**	8.63E-04	4.36E-02

ENSG00000139973	**SYT16**	synaptotagmin 16	**1.64**	1.03E-05	8.87E-05	**2.18**	7.40E-06	3.15E-03
ENSG00000149573	**MPZL2**	myelin protein zero like 2	**1.76**	1.25E-15	6.59E-14	**2.1**	9.23E-04	4.46E-02
ENSG00000112299	**VNN1**	vanin 1	**3.89**	5.11E-27	9.55E-25	**1.92**	8.13E-04	4.27E-02
ENSG00000187323	**DCC**	DCC netrin 1 receptor	**2.42**	4.19E-27	7.97E-25	**1.83**	1.62E-04	1.85E-02
ENSG00000131171	**SH3BGRL**	SH3 domain binding glutamate rich protein like	**2.08**	1.17E-51	1.69E-48	**1.83**	2.76E-05	6.69E-03
ENSG00000188404	**SELL**	selectin L	**1.93**	4.52E-07	5.23E-06	**1.77**	8.70E-04	4.36E-02
ENSG00000176463	**SLCO3A1**	solute carrier organic anion transporter family member 3A1	**2.41**	4.61E-05	3.36E-04	**1.68**	1.55E-04	1.82E-02
ENSG00000198157	**HMGN5**	high mobility group nucleosome binding domain 5	**2.48**	2.88E-44	2.50E-41	**1.68**	3.10E-04	2.53E-02
ENSG00000043591	**ADRB1**	adrenoceptor beta 1	**2.38**	1.54E-39	9.06E-37	**1.67**	1.85E-04	2.00E-02
ENSG00000169740	**ZNF32**	zinc finger protein 32	**2.55**	5.31E-39	3.03E-36	**1.55**	3.51E-04	2.67E-02
ENSG00000120885	**CLU**	microRNA 6843	**2.53**	4.85E-40	3.00E-37	**1.42**	2.71E-05	6.69E-03
ENSG00000087842	**PIR**	pirin	**2.49**	5.23E-37	2.58E-34	**1.41**	8.68E-04	4.36E-02
ENSG00000138829	**FBN2**	fibrillin 2	**1.52**	6.65E-21	6.71E-19	**1.35**	1.17E-03	4.97E-02
ENSG00000147408	**CSGALNACT1**	chondroitin sulfate N-acetyl-galactosaminyl-transferase 1	**2.75**	3.80E-40	2.42E-37	**1.34**	9.36E-05	1.33E-02
ENSG00000197410	**DCHS2**	dachsous cadherin-related 2	**1.92**	3.79E-03	1.55E-02	**1.33**	8.04E-04	4.24E-02
ENSG00000178343	**SHISA3**	shisa family member 3	**1.72**	2.19E-20	2.11E-18	**1.31**	6.93E-08	1.02E-04
ENSG00000081923	**ATP8B1**	ATPase phospholipid transporting 8B1	**2.56**	1.94E-36	8.75E-34	**1.28**	1.92E-04	2.04E-02
ENSG00000169760	**NLGN1**	neuroligin 1	**1.82**	1.32E-20	1.29E-18	**1.15**	4.89E-05	9.49E-03
ENSG00000187764	**SEMA4D**	semaphorin 4D	**2**	4.21E-06	3.96E-05	**1.12**	2.98E-04	2.53E-02
ENSG00000168077	**SCARA3**	scavenger receptor class A member 3	**1.52**	7.16E-25	1.15E-22	**1**	5.75E-04	3.54E-02
ENSG00000164733	**CTSB**	cathepsin B	**1.59**	6.49E-35	2.41E-32	**0.86**	9.60E-04	4.57E-02

Table S3B: Genes significantly and reproducibly down-regulated in FGFRL1-KD cells – xenografts and 2D monolayer cultures, corresponding to Venn diagram shown in Fig. 5C.

ENSEMBL GENE_ID	SYMBOL	GENE NAME	logFC xenografts	p-value	p-value adjusted	logFC cell line	p-value	p-value adjusted
ENSG00000166165	**CKB**	creatine kinase B	**-1.56**	1.71E-19	1.50E-17	**-0.97**	3.07E-05	6.87E-03
ENSG00000124615	**MOCS1**	molybdenum cofactor synthesis 1	**-2.23**	1.69E-14	7.54E-13	**-1.16**	7.78E-04	4.19E-02
ENSG00000142173	**COL6A2**	collagen type VI alpha 2 chain	**-2.01**	3.60E-36	1.53E-33	**-1.67**	4.40E-04	3.02E-02
ENSG00000181885	**CLDN7**	claudin 7	**-1.87**	4.60E-10	1.00E-08	**-1.72**	5.65E-04	3.52E-02
ENSG00000121653	**MAPK8IP1**	mitogen-activated protein kinase 8 interacting protein 1	**-1.57**	7.89E-31	2.17E-28	**-1.77**	4.79E-04	3.16E-02
ENSG00000165186	**PTCHD1**	patched domain containing 1	**-2.65**	1.87E-53	3.70E-50	**-2**	4.37E-04	3.02E-02
ENSG00000078114	**NEBL**	nebulette	**-1.84**	1.30E-25	2.18E-23	**-2.03**	1.32E-05	4.27E-03
ENSG00000184292	**TACSTD2**	tumor-associated calcium signal transducer 2	**-2.84**	2.42E-21	2.56E-19	**-2.05**	3.37E-04	2.59E-02
ENSG00000182389	**CACNB4**	calcium voltage-gated channel auxiliary subunit beta 4	**-3.07**	1.75E-05	1.42E-04	**-2.47**	8.42E-06	3.47E-03
ENSG00000141526	**SLC16A3**	Solute carrier 16 A 3	**-1.71**	3.47E-40	2.28E-37	**-2.48**	4.82E-04	3.16E-02
ENSG00000150051	**MKX**	mohawk homeobox	**-2.56**	1.35E-36	6.35E-34	**-2.62**	5.24E-05	9.71E-03
ENSG00000157833	**GAREM2**	GRB2 associated regulator of MAPK1 subtype 2	**-2.25**	6.42E-16	3.51E-14	**-2.74**	6.34E-04	3.77E-02
ENSG00000116016	**EPAS1**	endothelial PAS domain protein 1	**-4.4**	5.03E-24	7.63E-22	**-2.97**	2.41E-04	2.29E-02
ENSG00000012171	**SEMA3B**	microRNA 6872	**-1.57**	3.06E-15	1.50E-13	**-3.03**	5.14E-04	3.31E-02
ENSG00000120784	**ZFP30**	ZFP30 zinc finger protein	**-8.96**	1.37E-21	1.55E-19	**-4.85**	7.97E-05	1.20E-02
ENSG00000198538	**ZNF28**	zinc finger protein 28	**-3.5**	4.05E-07	4.74E-06	**-4.89**	6.43E-04	3.78E-02
ENSG00000244219	**TMEM225B**	TMEM225B	**-8.05**	4.10E-17	2.69E-15	**-6.01**	1.06E-05	3.79E-03
ENSG00000203995	**ZYG11A**	zyg-11 family member A, cell cycle regulator	**-8.88**	9.29E-21	9.28E-19	**-6.22**	1.20E-05	4.13E-03
ENSG00000179542	**SLITRK4**	SLIT and NTRK like family member 4	**-7.31**	1.50E-28	3.36E-26	**-7.21**	7.68E-04	4.16E-02

Table S4A: Top 100 most prominently up-regulated genes in mouse xenograft tumors after stable knock-down of FGFRL1.

SYMBOL	ENSEMBL_GENE_ID	Gene Name (ENSEMBL)	log2 Fold Change	p-value adjusted
**TMPRSS3**	ENSG00000160183	transmembrane serine protease 3	**11.53**	1.81E-30
**CNNM1**	ENSG00000119946	cyclin and CBS domain divalent metal cation transport mediator 1	**10.51**	3.73E-23
**ATF7IP2**	ENSG00000166669	activating transcription factor 7 interacting protein 2	**9.56**	4.68E-19
**MAGEB16**	ENSG00000189023	MAGE family member B16	**9.56**	2.76E-17
**BMP7**	ENSG00000101144	bone morphogenetic protein 7	**9.02**	3.19E-17
**PDPN**	ENSG00000162493	podoplanin	**8.80**	2.27E-12
**NCAM1**	ENSG00000149294	neural cell adhesion molecule 1	**8.34**	1.75E-03
**FAM155B**	ENSG00000130054	family with sequence similarity 155 B	**8.19**	4.71E-08
**ZNF536**	ENSG00000198597	zinc finger protein 536	**8.18**	1.91E+01
**LRP3**	ENSG00000130881	LDL receptor related protein 3	**8.14**	6.87E-10
**PHACTR3**	ENSG00000087495	phosphatase and actin regulator 3	**8.01**	2.58E-30
**LARGE1**	ENSG00000133424	LARGE xylosyl- and glucuronyltransfer. 1	**8.00**	7.06E-01
**SLFN11**	ENSG00000172716	schlafen family member 11	**7.92**	4.91E+01
**MGAT4A**	ENSG00000071073	alpha-1,3-mannosyl-glycoprotein 4-beta-N-acetylglucosaminyltransferase A	**7.91**	6.59E-25
**TBX5-AS1**	ENSG00000255399	TBX5 antisense RNA 1	**7.82**	2.75E+01
**GOLGA8G**	ENSG00000183629	golgin A8 family member G	**7.79**	1.41E-08
**ANKRD30B**	ENSG00000180777	ankyrin repeat domain 30B	**7.79**	3.66E-03
**FGF23**	ENSG00000118972	fibroblast growth factor 23	**7.77**	3.55E-07
**AR**	ENSG00000169083	androgen receptor	**7.77**	6.67E-06
**COL22A1**	ENSG00000169436	collagen type XXII alpha 1 chain	**7.68**	5.21E-02
**CRMP1**	ENSG00000072832	collapsin response mediator protein 1	**7.67**	8.35E-01
**DLGAP2**	ENSG00000198010	DLG associated protein 2	**7.64**	2.13E-01
**TIMP3**	ENSG00000100234	TIMP metallopeptidase inhibitor 3	**7.60**	1.52E+00
**LPL**	ENSG00000175445	lipoprotein lipase	**7.52**	2.10E-01
**FOXF1**	ENSG00000103241	forkhead box F1	**7.42**	4.47E-05
**CXADRP3**	ENSG00000265766	CXADR pseudogene 3	**7.42**	1.27E+02
**PPP1R14C**	ENSG00000198729	protein phosphatase 1 regulatory inhibitor subunit 14C	**7.29**	2.31E-05
**LRIG1**	ENSG00000144749	leucine rich repeats and immunoglobulin like domains 1	**7.17**	2.07E-11
**TBX5**	ENSG00000089225	T-box transcription factor 5	**7.06**	4.15E+00
**ROR2**	ENSG00000169071	receptor tyrosine kinase like orphan rec. 2	**6.96**	1.25E-01
**GABRA5**	ENSG00000186297	gamma-aminobutyric acid type A receptor alpha5 subunit	**6.96**	1.99E-02

**CLRN3**	ENSG00000180745	clarin 3	**6.95**	8.03E+00
**NDRG2**	ENSG00000165795	NDRG family member 2	**6.92**	1.44E+01
**WNT3A**	ENSG00000154342	Wnt family member 3A	**6.89**	1.41E+01
**OR10J3**	ENSG00000196266	olfactory receptor family 10 subfamily J member 3	**6.89**	6.97E-05
**LINC00993**	ENSG00000235687	long intergenic non-protein coding RNA 993	**6.86**	2.10E-04
**FAM189A1**	ENSG00000104059	family with sequence similarity 189 member A1	**6.84**	2.34E+00
**CECR2**	ENSG00000099954	CECR2 histone acetyl-lysine reader	**6.79**	1.73E+01
**TMEM132D-AS1**	ENSG00000249196	TMEM132D antisense RNA 1	**6.78**	5.14E-03
**TSHZ2**	ENSG00000182463	teashirt zinc finger homeobox 2	**6.72**	2.68E-04
**EMP2**	ENSG00000213853	epithelial membrane protein 2	**6.61**	3.12E-43
**CSF2RA**	ENSG00000198223	colony stimulating factor 2 receptor alpha subunit	**6.58**	2.33E-07
**LITAF**	ENSG00000189067	lipopolysaccharide induced TNF factor	**6.57**	2.29E-03
**TFAP2C**	ENSG00000087510	transcription factor AP-2 gamma	**6.56**	4.60E-03
**CHST15**	ENSG00000182022	carbohydrate sulfotransferase 15	**6.56**	1.60E-03
**LDLRAD4**	ENSG00000168675	low density lipoprotein receptor class A domain containing 4	**6.55**	2.69E-03
**CRNDE**	ENSG00000245694	colorectal neoplasia differentially expressed	**6.54**	4.10E-11
**IRX5**	ENSG00000176842	iroquois homeobox 5	**6.53**	3.73E-05
**SYT8**	ENSG00000149043	synaptotagmin 8	**6.51**	9.75E+00
**MAGEA5**	ENSG00000242520	MAGE family member A5	**6.49**	5.75E-05
**IRX4**	ENSG00000113430	iroquois homeobox 4	**6.48**	5.78E-03
**TMC3-AS1**	ENSG00000259343	TMC3 antisense RNA 1	**6.43**	2.49E-04
**ADARB2**	ENSG00000185736	adenosine deaminase RNA specific B2 (inactive)	**6.41**	5.79E-02
**ATRNL1**	ENSG00000107518	attractin like 1	**6.37**	2.25E-16
**FZD7**	ENSG00000155760	frizzled class receptor 7	**6.35**	2.30E-16
**MYO18B**	ENSG00000133454	myosin XVIIIB	**6.35**	3.43E-01
**FAM20C**	ENSG00000177706	FAM20C golgi associated secretory pathway kinase	**6.34**	7.01E-01
**LPAR4**	ENSG00000147145	lysophosphatidic acid receptor 4	**6.26**	7.82E-07
**DIRAS2**	ENSG00000165023	DIRAS family GTPase 2	**6.25**	2.57E-03
**EZHIP**	ENSG00000187690	EZH inhibitory protein	**6.25**	2.21E-02
**SNTG2**	ENSG00000172554	syntrophin gamma 2	**6.23**	1.02E-02
**MAN1C1**	ENSG00000117643	mannosidase alpha class 1C member 1	**6.22**	3.70E-03
**LRRC38**	ENSG00000162494	leucine rich repeat containing 38	**6.20**	1.89E-01
**ADGRE1**	ENSG00000174837	adhesion G protein-coupled receptor E1	**6.19**	5.84E-16
**XKR3**	ENSG00000172967	XK related 3	**6.17**	3.31E-04
**TPO**	ENSG00000115705	thyroid peroxidase	**6.14**	3.16E-01
**ADAMTS16**	ENSG00000145536	ADAM metallopeptidase with thrombospondin type 1 motif 16	**6.12**	2.28E-01

**H19**	ENSG00000130600	H19 imprinted maternally expressed transcript	**6.11**	2.33E-07
**CRYZL2P**	ENSG00000242193	crystallin zeta like 2, pseudogene	**6.10**	3.15E-02
**EMX1**	ENSG00000135638	empty spiracles homeobox 1	**6.08**	2.28E+02
**SDK1**	ENSG00000146555	sidekick cell adhesion molecule 1	**6.07**	4.80E+00
**KAZN**	ENSG00000189337	kazrin, periplakin interacting protein	**6.02**	3.52E-01
**MNX1-AS1**	ENSG00000243479	MNX1 antisense RNA 1 (head to head)	**6.02**	3.82E-14
**LILRA2**	ENSG00000239998	leukocyte immunoglobulin like receptor A2	**5.98**	2.53E-01
**UBASH3A**	ENSG00000160185	ubiquitin associated and SH3 domain containing A	**5.95**	5.75E-02
**RIMKLB**	ENSG00000166532	ribosomal modification protein rimK like family member B	**5.90**	2.39E-01
**TRIM22**	ENSG00000132274	tripartite motif containing 22	**5.88**	3.31E-02
**SHANK2**	ENSG00000162105	SH3 and multiple ankyrin repeat domains 2	**5.88**	1.03E+02
**FOXI1**	ENSG00000168269	forkhead box I1	**5.87**	1.34E+01
**LINC02152**	ENSG00000261319	long intergenic non-protein coding RNA 2152	**5.86**	5.66E-01
**TESC**	ENSG00000088992	tescalcin	**5.85**	4.87E-01
**GAS7**	ENSG00000007237	growth arrest specific 7	**5.84**	4.28E+02
**LINC01257**	ENSG00000204603	long intergenic non-protein coding RNA 1257	**5.83**	1.80E+00
**ERO1B**	ENSG00000086619	endoplasmic reticulum oxidoreductase 1 beta	**5.78**	5.29E-20
**CYTL1**	ENSG00000170891	cytokine like 1	**5.78**	1.30E+00
**C7orf33**	ENSG00000170279	chromosome 7 open reading frame 33	**5.77**	1.88E+00
**THBS2**	ENSG00000186340	thrombospondin 2	**5.71**	1.80E-09
**ZNF516**	ENSG00000101493	zinc finger protein 516	**5.70**	5.07E-06
**LINC01484**	ENSG00000253686	long intergenic non-protein coding RNA 1484	**5.70**	6.00E-02
**BRINP1**	ENSG00000078725	BMP/retinoic acid inducible neural specific 1	**5.64**	1.35E-06
**PIEZO2**	ENSG00000154864	piezo type mechanosensitive ion channel component 2	**5.61**	1.62E-01
**ZNF157**	ENSG00000147117	zinc finger protein 157	**5.57**	6.19E+00
**NKD1**	ENSG00000140807	NKD inhibitor of WNT signaling pathway 1	**5.57**	6.60E-01
**NAP1L3**	ENSG00000186310	nucleosome assembly protein 1 like 3	**5.57**	4.59E-33
**EYA2**	ENSG00000064655	EYA transcriptional coactivator and phosphatase 2	**5.56**	2.92E-02
**ZMAT1**	ENSG00000166432	zinc finger matrin-type 1	**5.55**	5.18E-11
**ZNF423**	ENSG00000102935	zinc finger protein 423	**5.55**	5.86E-05

**PTX3**	ENSG00000163661	pentraxin 3	**5.55**	6.47E-18
**SPATA48**	ENSG00000164500	spermatogenesis associated 48	**5.55**	3.75E+00
**SDC2**	ENSG00000169439	syndecan 2	**5.54**	4.83E-01

Table S4B: Top 100 most prominently down-regulated genes in mouse xenograft tumors after stable knock-down of FGFRL1.

SYMBOL	ENSEMBL_GENE_ID	Gene Name (ENSEMBL)	log2 Fold Change	p-value adjusted
**TMEM168**	ENSG00000146802	transmembrane protein 168	**-10.90**	8.10E-26
**ZFP30**	ENSG00000120784	ZFP30 zinc finger protein	**-8.96**	1.55E-15
**ZYG11A**	ENSG00000203995	zyg-11 family member A, cell cycle regulator	**-8.88**	9.28E-15
**SMO**	ENSG00000128602	smoothened, frizzled class receptor	**-8.31**	1.89E-35
**MIR548XHG**	ENSG00000224141	MIR548X host gene	**-8.10**	6.51E-11
**TMEM225B**	ENSG00000244219	transmembrane protein 225B	**-8.05**	2.69E-11
**SERPINB9**	ENSG00000170542	serpin family B member 9	**-7.88**	4.03E-10
**ZSCAN18**	ENSG00000121413	zinc finger and SCAN domain containing 18	**-7.83**	3.44E-13
**SLITRK4**	ENSG00000179542	SLIT and NTRK like family member 4	**-7.31**	3.36E-22
**PTGS2**	ENSG00000073756	prostaglandin-endoperoxide synthase 2	**-6.81**	5.65E-06
**SLC26A4-AS1**	ENSG00000233705	SLC26A4 antisense RNA 1	**-6.30**	1.29E-03
**PPDPFL**	ENSG00000168333	pancreatic progenitor cell differentiation and proliferation factor like	**-6.19**	4.44E-14
**CXCL1**	ENSG00000163739	C-X-C motif chemokine ligand 1	**-6.19**	3.87E-03
**CLDN10-AS1**	ENSG00000223392	CLDN10 antisense RNA 1	**-6.14**	3.77E-04
**RAB6B**	ENSG00000154917	RAB6B, member RAS oncogene family	**-6.14**	1.41E-65
**DNAJC15**	ENSG00000120675	DnaJ heat shock protein family (Hsp40) member C15	**-5.93**	9.72E-05
**CD79B**	ENSG00000007312	CD79b molecule	**-5.86**	1.16E-02
**ZNF347**	ENSG00000197937	zinc finger protein 347	**-5.75**	6.91E-02
**IL1RAPL1**	ENSG00000169306	interleukin 1 receptor accessory protein like 1	**-5.72**	1.08E-02
**ENG**	ENSG00000106991	endoglin	**-5.70**	8.47E-02
**LINC02820**	ENSG00000258815	long intergenic non-protein coding RNA 2820	**-5.63**	2.16E-01
**KCNH5**	ENSG00000140015	potassium voltage-gated channel subfamily H member 5	**-5.54**	3.39E+00
**LAG3**	ENSG00000089692	lymphocyte activating 3	**-5.51**	1.78E-159
**ZNF607**	ENSG00000198182	zinc finger protein 607	**-5.44**	1.97E-03
**SLC10A4**	ENSG00000145248	solute carrier family 10 member 4	**-5.36**	5.74E+00
**BCL2A1**	ENSG00000140379	BCL2 related protein A1	**-5.12**	1.36E+01

**ZNF256**	ENSG00000152454	zinc finger protein 256	**-5.09**	1.63E-15
**RPS6KL1**	ENSG00000198208	ribosomal protein S6 kinase like 1	**-5.05**	1.89E+00
**FGG**	ENSG00000171557	fibrinogen gamma chain	**-4.87**	3.34E+01
**IGFBP3**	ENSG00000146674	insulin like growth factor binding protein 3	**-4.85**	7.80E-20
**OTOGL**	ENSG00000165899	otogelin like	**-4.84**	7.24E-05
**CDH5**	ENSG00000179776	cadherin 5	**-4.84**	1.54E+01
**LINC01357**	ENSG00000224167	long intergenic non-protein coding RNA 1357	**-4.81**	1.20E-09
**TMPRSS15**	ENSG00000154646	transmembrane serine protease 15	**-4.73**	2.01E-18
**XIST**	ENSG00000229807	X inactive specific transcript	**-4.71**	6.74E+00
**C6orf223**	ENSG00000181577	chromosome 6 open reading frame 223	**-4.70**	2.89E-03
**DAAM2**	ENSG00000146122	dishevelled associated activator of morphogenesis 2	**-4.64**	4.10E+01
**MT1M**	ENSG00000205364	metallothionein 1M	**-4.62**	1.09E+02
**OGN**	ENSG00000106809	osteoglycin	**-4.57**	8.01E-02
**CDC42P2**	ENSG00000234844	cell division cycle 42 pseudogene 2	**-4.57**	1.69E+01
**SYN1**	ENSG00000008056	synapsin I	**-4.48**	2.40E+01
**HPN**	ENSG00000105707	hepsin	**-4.46**	1.25E-01
**UGT2B10**	ENSG00000109181	UDP glucuronosyltransferase family 2 member B10	**-4.41**	2.63E-01
**PRG4**	ENSG00000116690	proteoglycan 4	**-4.40**	1.75E-03
**EPAS1**	ENSG00000116016	endothelial PAS domain protein 1	**-4.40**	7.63E-18
**NXF3**	ENSG00000147206	nuclear RNA export factor 3	**-4.36**	2.54E-02
**TNFSF14**	ENSG00000125735	TNF superfamily member 14	**-4.35**	2.89E+00
**SCNN1G**	ENSG00000166828	sodium channel epithelial 1 gamma subunit	**-4.35**	1.66E-13
**LRRK2**	ENSG00000188906	leucine rich repeat kinase 2	**-4.34**	1.25E-03
**ACSL5**	ENSG00000197142	acyl-CoA synthetase long chain family member 5	**-4.33**	1.70E-05
**CYP2C18**	ENSG00000108242	cytochrome P450 family 2 subfamily C member 18	**-4.32**	1.30E+02
**TREM1**	ENSG00000124731	triggering receptor expressed on myeloid cells 1	**-4.31**	7.07E-04
**AQP1**	ENSG00000240583	aquaporin 1 (Colton blood group)	**-4.26**	2.86E-13
**EVI2A**	ENSG00000126860	ecotropic viral integration site 2A	**-4.26**	4.47E+01
**PDZK1IP1**	ENSG00000162366	PDZK1 interacting protein 1	**-4.24**	2.18E+01
**SLC35G2**	ENSG00000168917	solute carrier family 35 member G2	**-4.21**	1.85E-25
**CDH13**	ENSG00000140945	cadherin 13	**-4.12**	6.42E-04
**TGM2**	ENSG00000198959	transglutaminase 2	**-4.11**	6.17E-05
**SCNN1B**	ENSG00000168447	sodium channel epithelial 1 beta subunit	**-4.11**	2.10E-05

**DENND2D**	ENSG00000162777	DENN domain containing 2D	**-4.09**	6.00E-53
**VWA1**	ENSG00000179403	von Willebrand factor A domain containing 1	**-4.07**	2.54E-02
**GJB4**	ENSG00000189433	gap junction protein beta 4	**-4.02**	8.94E+01
**PDE8B**	ENSG00000113231	phosphodiesterase 8B	**-3.99**	1.95E-15
**RSPO2**	ENSG00000147655	R-spondin 2	**-3.98**	3.08E+02
**ACKR3**	ENSG00000144476	atypical chemokine receptor 3	**-3.95**	2.11E-11
**CSF2**	ENSG00000164400	colony stimulating factor 2	**-3.94**	7.82E+00
**SIRPG-AS1**	ENSG00000237914	SIRPG antisense RNA 1	**-3.93**	3.22E+02
**LINC01714**	ENSG00000227634	long intergenic non-protein coding RNA 1714	**-3.92**	2.56E+02
**ANKRD60**	ENSG00000124227	ankyrin repeat domain 60	**-3.86**	7.11E-06
**NDUFA4L2**	ENSG00000185633	NDUFA4 mitochondrial complex associated like 2	**-3.85**	3.05E-06
**HHLA2**	ENSG00000114455	HERV-H LTR-associating 2	**-3.85**	2.09E-01
**AGTR1**	ENSG00000144891	angiotensin II receptor type 1	**-3.84**	6.87E+01
**LGI2**	ENSG00000153012	leucine rich repeat LGI family member 2	**-3.83**	1.80E-11
**GFAP**	ENSG00000131095	glial fibrillary acidic protein	**-3.71**	3.05E+02
**ELOVL3**	ENSG00000119915	ELOVL fatty acid elongase 3	**-3.69**	2.89E+02
**C6orf132**	ENSG00000188112	chromosome 6 open reading frame 132	**-3.68**	1.34E-02
**ASB14**	ENSG00000239388	ankyrin repeat and SOCS box containing 14	**-3.67**	3.46E+02
**RTL4**	ENSG00000187823	retrotransposon Gag like 4	**-3.65**	2.32E-02
**CLDN24**	ENSG00000185758	claudin 24	**-3.64**	5.84E-02
**P4HA3-AS1**	ENSG00000246211	P4HA3 antisense RNA 1	**-3.62**	3.99E+02
**FGB**	ENSG00000171564	fibrinogen beta chain	**-3.60**	1.26E+01
**ZNF518A**	ENSG00000177853	zinc finger protein 518A	**-3.59**	3.24E+02
**CD86**	ENSG00000114013	CD86 molecule	**-3.59**	9.20E-02
**OASL**	ENSG00000135114	2'-5'-oligoadenylate synthetase like	**-3.57**	1.48E-11
**CSPG4**	ENSG00000173546	chondroitin sulfate proteoglycan 4	**-3.56**	1.47E+01
**SCGB1D2**	ENSG00000124935	secretoglobin family 1D member 2	**-3.56**	4.53E+02
**FAM225A**	ENSG00000231528	family with sequence similarity 225 member A	**-3.56**	1.83E+01
**PLA2G3**	ENSG00000100078	phospholipase A2 group III	**-3.55**	9.34E+00
**ANO3**	ENSG00000134343	anoctamin 3	**-3.55**	7.61E+00
**CPA5**	ENSG00000158525	carboxypeptidase A5	**-3.50**	2.16E+02
**ZNF28**	ENSG00000198538	zinc finger protein 28	**-3.50**	4.74E-02
**LINC01356**	ENSG00000215866	long intergenic non-protein coding RNA 1356	**-3.49**	2.43E+00
**KCNJ15**	ENSG00000157551	potassium voltage-gated channel subfamily J member 15	**-3.45**	8.01E-02
**CCN4**	ENSG00000104415	cellular communication network factor 4	**-3.44**	3.10E-30

**ANGPTL4**	ENSG00000167772	angiopoietin like 4	**-3.44**	1.90E-07
**FREM1**	ENSG00000164946	FRAS1 related extracellular matrix 1	**-3.43**	8.18E-01
**NYNRIN**	ENSG00000205978	NYN domain and retroviral integrase containing	**-3.38**	2.55E-02
**TINAGL1**	ENSG00000142910	tubulointerstitial nephritis antigen like 1	**-3.37**	1.75E-03
**SPTLC3**	ENSG00000172296	serine palmitoyltransferase long chain base subunit 3	**-3.32**	3.91E+00
**TBPL2**	ENSG00000182521	TATA-box binding protein like 2	**-3.29**	4.38E+02

Table S5: Significantly enriched tumor/stroma interactions detected by the CASTIN algorithm in PC-3M xenografts derived from FGFRL1-KD versus control cells.

GENE SYMBOLS	MEAN Tumor (human)	MEAN Stroma (mouse)	SD Tumor	SD Stroma	P-VALUE
**Enriched in PC-3M xenografts**					
SEMA4F (tumor) / NRP2 (stroma)	1.121	0.987	0.028	0.026	0.0001
COL2A1 (tumor) / ITGA2B (stroma)	0.031	-0.644	0.057	0.174	0.003
VEGFC (tumor) / FLT4 (stroma)	1.243	1.481	0.122	0.075	0.009
FGF23 (tumor) / FGFR3 (stroma)	0.101	-0.593	0.304	0.080	0.015
EFNB3 (tumor) / EPHB3 (stroma)	0.669	0.386	0.147	0.097	0.018
FGF2 (tumor) / FGFR3 (stroma)	0.593	0.360	0.111	0.089	0.028
CCL26 (tumor) / CCR1 (stroma)	1.206	1.377	0.112	0.094	0.032
JAG2 (tumor) / NOTCH3 (stroma)	1.304	1.427	0.052	0.088	0.034
DKK1 (tumor) / KREMEN1 (stroma)	2.290	2.132	0.086	0.106	0.034
FGF2 (tumor) / GPC4 (stroma)	1.193	0.965	0.029	0.087	0.034
EGF (tumor) / EGFR (stroma)	0.907	0.723	0.113	0.013	0.045
VEGFC (tumor) / KDR (stroma)	1.568	1.719	0.055	0.099	0.048
**Enriched in stromal (mouse) tissue**					
NTN1 (stroma) / DCC (tumor)	0.903	0.421	0.104	0.116	0.0001
ADAM12 (stroma) / EGF (tumor)	1.214	0.928	0.047	0.029	0.0002
LAMA1 (stroma) / ITGA2 (tumor)	0.998	0.747	0.113	0.084	0.005
EPHB3 (stroma) / EFNB3 (tumor)	0.669	0.386	0.147	0.097	0.018
TNFSF10 (stroma) / TNFRSF10C (tumor)	0.509	0.182	0.042	0.195	0.018
INHBA (stroma) / ACVR1B (tumor)	1.311	1.552	0.090	0.092	0.018

Stroma: corresponds to reads matched to the mouse genome (= mouse stromal cells in xenografts)
Tumour: corresponds to reads matched to human genome (= PC3M cells in xenografts)

Table S6: The sequencing coverage and quality statistics of next generation sequencing (NGS) data for PC3M control and FGFRL1 knock down cell line and xenografts.

Sample set*	Sample ID	RNA integrity number (RIN)	Total number of sequenced reads	Total number of uniquely mapped reads	Ratio of all reads aligned to rRNA regions to total uniquely mapped reads (rRNA rate) [%]	Ratio of exon-mapped rea ds to total uniquely mapped reads (Expression Profile Efficiency) [%]	Total number of de tected transcripts with reads ≥1	Reference genome
I	21_492_ctrl_sh5 (PC3M control clone 5)	9.5	9771694	8214742	0.2	33.5	25375	GRCh38.81
I	22_493_ctrl_sh5 (PC3M control clone 5)	10	7335684	6153615	0.3	33.9	23575	GRCh38.81
I	23_609_ctrl_sh8 (PC3M control clone 8)	10	14943012	12643362	0.1	32.9	27798	GRCh38.81
I	24_610_ctrl_sh8 (PC3M control clone 8)	10	14578922	12418712	0.1	32.9	27909	GRCh38.81
I	33_615_FGFR5sh11 (PC3M KD clone 11)	10	8407203	7164026	0.1	32.8	21871	GRCh38.81
I	34_616_FGFR5sh11 (PC3M KD clone 11)	10.0	9660825	8216287	0.1	32.7	23394	GRCh38.81
II	T1-5 (PC3M xenograft control)	9.4	23608595	16479360	2.1	78.0	34155	hg19
II	T1-6 (PC3M xenograft control)	9.7	36899072	26794663	1.9	78.8	34583	hg19
II	T2-1 (PC3M xenograft control)	9.5	26237705	19159521	2.1	80.2	34295	hg19
II	T2-3 (PC3M xenograft control)	9.6	48997888	35939600	1.8	79.0	34182	hg19
II	T2-4 (PC3M xenograft control)	9.9	40784423	29528974	1.6	78.0	35128	hg19
II	T5-2 (PC3M KD xenograft)	9.5	21535877	14669665	1.6	78.1	35168	hg19
II	T5-5 (PC3M KD xenograft)	9.7	37293152	26795537	1.9	77.3	34614	hg19
II	T6-1 (PC3M KD xenograft)	8.7	19846462	10891409	3.8	75.4	34222	hg19
II	T6-4 (PC3M KD xenograft)	9.5	27343702	19357981	1.7	77.1	35757	hg19
II	T6-5 (PC3M KD xenograft)	9.8	22550288	16665003	1.5	79.0	35130	hg19
*Set I, cell lines; Set II, xenografts								
